# How We Simulate Nanopores

**DOI:** 10.1002/smtd.70904

**Published:** 2026-08-03

**Authors:** Behzad Mehrafrooz, Xavier Mleziva, Meng Xu, Melika Shekari, Christopher Maffeo, Aleksei Aksimentiev

**Affiliations:** ^1^ Center for Biophysics and Quantitative Biology Urbana Illinois USA; ^2^ Beckman Institute for Advanced Science and Technology Urbana Illinois USA; ^3^ Department of Physics University of Illinois at Urbana–Champaign Urbana Illinois USA

**Keywords:** ionic current, membrane transport, molecular dynamics, nanopores, sensing, sequencing, simulation

## Abstract

Nanopore technology has revolutionized single‐molecule science by enabling ultrasensitive characterization of biomolecular structure and interactions via accessible, physics‐based tabletop measurements. In a typical experiment, the chemical structure of a biomolecule is determined by measuring a transient blockade of the nanopore ionic current produced by the nanopore passage of the molecule. Nanopore sensing can now deliver a telomere‐to‐telomere sequence of a human chromosome and is poised to unravel the vast complexity of the proteome. Notwithstanding the three decades of development, a fundamental interpretive bottleneck remains: the method cannot independently determine the 3D structure of a biomolecule as it passes through the nanopore. All‐atom molecular dynamics has emerged as a go‐to method for determining the structure of the translocating molecules and its relationship with the nanopore current. Here, we review molecular dynamics methods used to simulate the transport through biological, solid‐state, and DNA origami nanopores. We highlight how advances in high‐performance computing and enhanced sampling are dismantling historical timescale barriers, allowing the simulations to achieve unprecedented experimental relevance. By detailing the path from system construction to the extraction of experimental observables, we illustrate how computational insights are transitioning from providing post‐hoc signal interpretation to enabling predictive, rational design of next‐generation biosensors.

## Graphical abstract

Nanopore technology has revolutionized single‐molecule sensing, yet it suffers from an inherent interpretive challenge. Molecular dynamics has emerged as a go‐to method for determining the relationship between the structure of translocating molecules and the nanopore current signals. This review describes the landscape of nanopore simulations, highlighting recent advances that have allowed the simulations to achieve unprecedented experimental relevance.

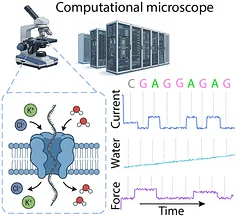

## 1 Introduction

Over the past three decades, the nanopore sensing principle [1] has transformed into a versatile platform for direct, label‐free electrical detection of biomolecular structure and interactions [2, 3]. While its development was initially driven by the quest toward a $1000 human genome [4, 5], having accomplished that [6, 7, 8], the scope of nanopore sensing has expanded to RNA [9, 10, 11], protein [12, 13, 14, 15] and glycan [16, 17, 18, 19] sensing and sequencing, small molecule sensing [20, 21], drug design [22, 23] and ion recognition and separation [24, 25, 26]. Fundamentally, the method relies on an externally applied electric field to drive ions and charged analytes through a nanoscale orifice, generating characteristic ionic current blockades [27] (Figure 1). Each capture event is characterized by a blockade amplitude, reflecting the fractional reduction of the open‐pore current, and by a dwell time, which reflects the residence time of the analyte within the sensing region. Additional observables such as the capture rate and intra‐event current switching encode information about molecular concentration, conformational heterogeneity, and binding kinetics (Figure 1d). For sufficiently long polymers, including nucleic acids and polypeptides, translocation produces continuous current traces (“squiggles”) composed of sequential blockade levels, each arising from distinct nucleotide or amino‐acid segments occupying the pore constriction. These temporal signatures encode rich multidimensional data regarding the size, chemical structure, charge distribution, and conformational dynamics of single molecules [28, 29, 30].

**FIGURE 1:**
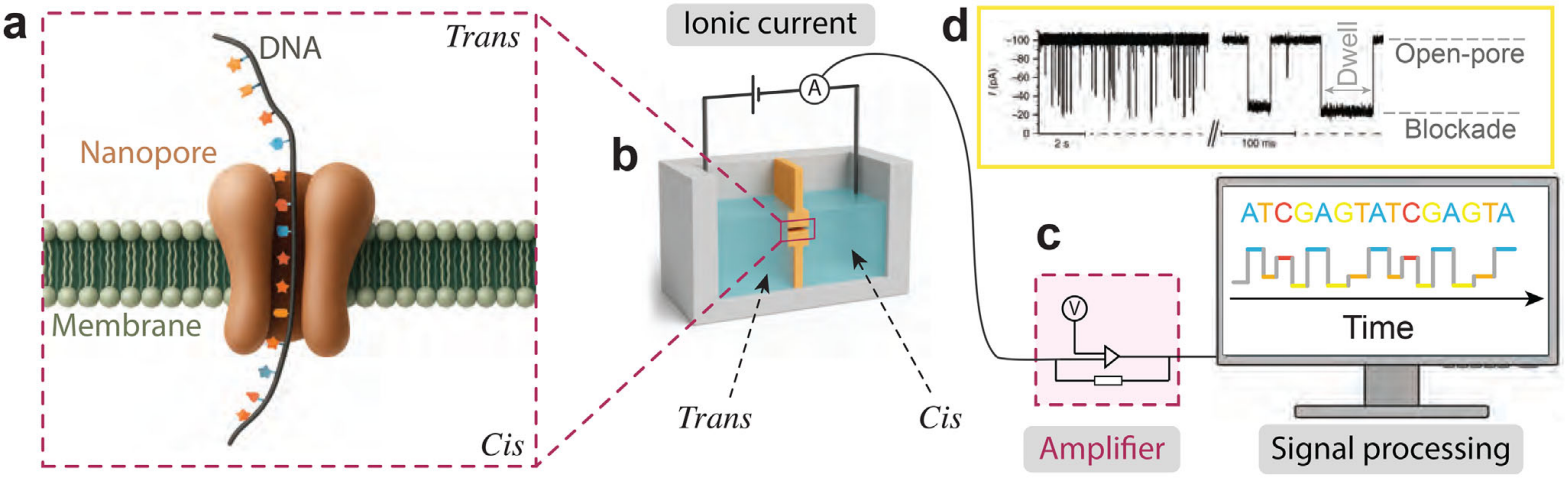
Principle of nanopore sensing of biomolecules. (a) A biological nanopore (orange) is embedded in a lipid membrane (green), separating the *cis* and *trans* chambers of an electrolytic cell. (b) Upon application of an electric potential across the membrane, ions flow through the nanopore, producing a steady open‐pore ionic current. When a biomolecule, here illustrated as single‐stranded DNA, enters and translocates through the pore, it partially obstructs the ionic pathway, leading to characteristic reductions of the current. (c) The ionic current is amplified and processed electronically, enabling real‐time monitoring of the encoded information regarding the chemical structure of the translocating analyte. (d) Representative ionic current trace recorded in a nanopore translocation experiment. In the trace, the open‐pore current is interspersed by transient blockade events each characterized by the blockade amplitude and the blockade duration (dwell time). Experimental data in panel d are adapted with permission from Piguet et al. [31], Copyright 2018, *Springer Nature* under a CC‐BY 4.0 license.

Despite the exquisite sensitivity of a nanopore measurement, the experimental readout comes with an inherent interpretive challenge: the inability to directly visualize the atomic‐scale conformations that govern signal generation. Because the ionic current serves as a one‐dimensional proxy for complex, three‐dimensional molecular dynamics, the causal link between a molecule's structure and its blockade signature often remains ambiguous. Thus, computational modeling has become an indispensable tool for resolving such ambiguity. While continuum descriptions based on mean‐field theories can efficiently estimate macroscopic ionic flux [32, 33, 34, 35, 36], they inherently lack the granular resolution required to describe specific solute‐pore interactions, hydration effects, and conformational changes. Operating at the scale of atoms, all‐atom molecular dynamics (MD) has emerged as the method of choice for decoding the microscopic physics of nanopore translocation [37, 38]. While the applications of the MD method to nanopore sensing problems were in the past tempered by the short timescales of the method and the high computational cost, recent advances in high‐performance computing and enhanced sampling algorithms are effectively dismantling these barriers. The near future MD simulations are expected to deliver nanopore translocation trajectories of unprecedented experimental relevance, offering an unadulterated, atomistic view of the dynamic interactions that define the nanopore sensing process.

In this review, we provide a framework for navigating the computational landscape of nanopore sensing, moving from system construction to the extraction of experimental observables. We begin by delineating the specific modeling requirements for the three dominant nanopore classes—biological proteins, solid‐state membranes, and DNA origami nanostructures—highlighting how system size and composition dictate the simulation strategy. We then scrutinize the technical execution of these simulations, identifying the critical forcefield parameters, water models, and integration algorithms necessary to avoid common artifacts in the simulations of nanopore transport. Next, we review the methods developed to simulate the analyte translocation through nanopore systems, as well as the methods to control nanopore transport. Finally, we describe the analytical tools used to connect the computationally generated trajectories with laboratory‐measured signals, focusing on the calculation of ionic current blockades, selectivity, and analyte translocation kinetics, ultimately providing a roadmap for converting computational insights into rational sensor design.

## 2 General Considerations

Nanopore systems can be represented using various models that can be placed on a scale of increasing complexity and computational cost, starting with continuum models and ending with all‐atom MD simulations. Models with less complexity may not provide a good description of biomolecular mechanics, represent ions explicitly or include hydrodynamic effects, potentially limiting their utility in nanopore studies where modulation of the ionic current due to interactions between individual ions and an analyte in the pore constriction are of significant interest. Hence, our preferred method is typically all‐atom MD, and most of the examples that follow will describe all‐atom MD simulations.

All models, including the all‐atom MD ones, are approximations, and it is important to understand the limitations of the model prior to its application. For example, MD employs a classical mechanics description and, unless using a suitable multiscale strategy (e.g., QM/MM [39]), is incapable of directly modeling certain phenomena including chemical reactions, redistribution of electrons in metals, semiconductors, carbon nanotubes and some 2D materials, and constant chemical potentials. Furthermore, all‐atom MD simulations are computationally costly, limiting simulations in practice to 100 nm length scales and sub‐millisecond timescales, with notable exceptions utilizing specialized hardware [40]. The limited length scales also imply a lower bound on the ion conditions that can be represented accurately, as the box dimensions should be several times the characteristic electrostatic screening length—the Debye length, which is ∼1 nm for 100 mM monovalent electrolyte and increases proportionally to the inverse of the square root of ionic strength. MD simulations of electrically charged objects at low salt conditions [41] are particularly challenging because the number of counterions needed to neutralize the object's charge should ideally be less than the number of counterions representing bulk electrolyte. Furthermore, the classical nature of all‐atom MD allows sufficiently large biases to be applied without causing dielectric breakdown of membrane materials. While such high‐bias simulations are useful for probing translocation scenarios and testing new simulation protocols, caution should be used when interpreting the outcome of such simulations to avoid physically impossible conditions for the materials present in a simulation. In general, MD algorithms are very robust and the fact that the simulation ran without crashing does not guarantee that the resulting MD trajectory is physically meaningful.

There are several common sense questions that one should answer before embarking on an all‐atom MD study. The most obvious one is: do we actually have sufficient information about the experiment to apply the all‐atom MD method? One such experiment is nanopore analysis of biological samples. In contrast to experiment, the all‐atom MD method operates with a pre‐determined composition of the simulation system so if some essential molecules are not included in the initial setup, they will not magically appear over the course of the MD run. Another class of difficult systems are nanopores of unknown structure. Typically, such systems exhibit one or several well‐defined conductance states. However, knowing the nanopore conductance is not sufficient to build an all‐atom model of the nanopore as multiple structural models can have the same conductance. One exception to that is conventional solid‐state nanopores, where knowing the overall pore geometry and the open pore conductance is often sufficient to build a useful MD model. Equally challenging are simulations of analytes whose structures are unknown. The advent of computational structure prediction tools has somewhat alleviated this problem, yet computational studies of partially disordered proteins remain challenging. A seemingly obvious consideration is the duration of a typical nanopore translocation event. While this indeed is an important factor for brute‐force MD considerations, enhanced sampling methods allow one to probe possible translocation scenarios without needing to run the simulation at the nominal experimental timescale.

For brute force simulations of a nanopore measurement, it is useful to consider the best case scenario where the ionic current signal is solely determined by the thermal noise. In such a scenario, the lower bound of the error for the ionic current determination can be computed for a given simulation duration from the experimental ionic current, which is the number of charges passing through the nanopore in a unit of time. The counting error for such a process goes as the inverse of the square root of the charge translocation events, $1/\sqrt{N}$. For example, a 160 pA current implies a passage of one elementary charge ($1.6\times 10^{-19}$ C) in one nanosecond. The relative error of the ionic current determination from a 10 ns simulation is then 33.3%, which becomes 10% for a 100 ns simulations and 3.3% for a microsecond trajectory. This simple “rule of thumb” provides an estimate for the minimal simulation duration required to determine an ionic current difference with a pre‐defined statistical error. In practice, however, additional sources of noise, such as the conformational dynamics of the analyte [42], can considerably increase the simulation time required to determine the ionic current to a specified precision. Nevertheless, the above considerations provide a common‐sense guidance to what can be expected from a brute force MD simulation. As we describe below, decoupling the simulation of analyte translocation from the ionic current determination circumvents the thermal noise problem of the brute force approach.

## 3 Computational Models of Nanopores

The landscape of nanopore sensing is defined by three primary architectural classes: biological, solid‐state, and DNA origami nanopores. Each category presents distinct physical properties and material compositions that dictate the specific computational strategies required to model them accurately. Biological nanopores, derived from protein assemblies, offer unparalleled atomic‐scale reproducibility and “programmability” through mutagenesis, yet they necessitate precise handling of protein‐lipid interactions and local chemical environments. In contrast, solid‐state nanopores are fabricated from inorganic materials like silicon nitride films or graphene, offering superior mechanical properties and a wider range of pore dimensions. MD simulations of such systems sometimes require custom models to account for the complex physics of the membrane materials and poorly characterized chemistry of the solid‐state surfaces exposed to electrolyte solutions. DNA origami nanopores utilize the specificity of Watson–Crick base pairing to provide highly tailored, large‐scale architectures that can be decorated with diverse chemical motifs.

Despite the remarkable progress in nanopore sensing technology, several practical bottlenecks remain for real‐world applications. Thus, protein nanopores can spontaneously and often reversibly change their structure in a process known as gating [43], which is sensitive to temperature, pH, electrolyte and mechanical stress, and their embedding lipid membranes are inherently fragile and prone to rupture [5]. DNA nanopores face additional challenges related to nuclease degradation in biological fluids and stability of their double‐stranded structure [44]. For solid‐state nanopores, surface fouling by nonspecific adsorption and gradual structural deterioration remain a persistent obstacle to long‐term reliable sensing [45].

In what follows, we review the specific modeling requirements and construction workflows for simulations of biological, solid‐state and DNA origami nanopores, highlighting recent developments that have improved the realism of such simulations.

### 3.1 Biological Nanopores

Biological nanopores are membrane‐spanning protein assemblies that form well‐defined, nanometer‐scale channels. Derived from bacterial toxins [46, 47], porins [48, 49], or transport proteins [50], biological nanopore monomers self‐assemble into remarkably stable multimeric architectures of high structural homogeneity [51]. These nanopores usually adopt a single, well‐defined conformation dictated by their primary sequence, ensuring that ionic current signatures and translocation kinetics remain highly reproducible across experiments [52]. While traditionally reconstituted into lipid bilayers, these pores can also be integrated into amphiphilic block‐copolymer membranes to improve the mechanical longevity of the nanopore sensor [53]. The functional anatomy of a biological nanopore can be conceptually divided into three general regions: the *vestibule*, the *constriction*, and the *lumen* (or a barrel) [44, 54] (Figure 2a). The vestibule is a wide, often conical opening that acts as a capture well, using long‐range electrostatic interactions to funnel analytes toward the transmembrane pore. The constriction is typically the narrowest point of the channel; it is here that the physical proximity between the pore walls and the analyte modulates the ionic current with the highest sensitivity. Finally, the lumen extends through the membrane, providing a confined environment where the side chains of the nanopore walls define the forces resisting the analytes transport and thus the residence time of the analyte within the nanopore. The versatility of the biological nanopores is further enhanced by their “programmability”. Through site‐directed mutagenesis or *de novo* protein design, researchers can precisely tune the physicochemical properties of these regions, for example, altering the charge of the vestibule to increase the analyte capture rate or modifying the hydrophobicity of the constriction to detect specific molecular motifs [55, 56]. This atomic‐scale configurability of biological nanopores allows for the creation of specialized sensors for everything from metal ions [57] and small metabolites [3] to genomic DNA [58] and folded proteins [59]. For a detailed overview of biological nanopore fabrication methods and applications, the reader is referred to the following reviews [44, 56, 60, 61].

**FIGURE 2:**
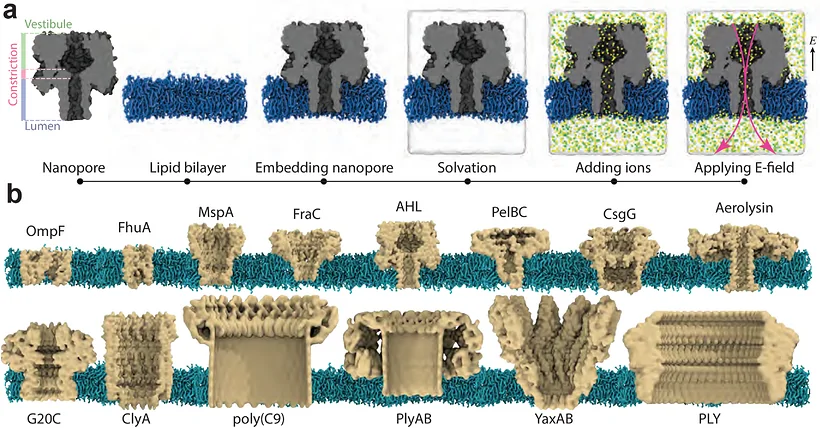
Construction of all‐atom nanopore systems and examples of biological nanopores. (a) Stepwise procedure for generating an all‐atom MD model of a nanopore system. Beginning with a pre‐equilibrated lipid bilayer, the nanopore structure is inserted, solvated with explicit water, ionized to the desired salt concentration, and subjected to an external electric field to drive ion flux and polymer translocation. (b) Most common biological nanopores used for nanopore sensing experiments: OmpF [62], FhuA [63, 64], MspA [65, 66], FraC [67], AHL [68], PelBC [69], CsgG [50], aerolysin [70], G20C [71], ClyA [72], poly(C9) [73], PlyAB [74], YaxAB [75], and PLY [76]. Each structure is depicted as a gold cutaway molecular surface embedded in a lipid bilayer (green); water and ions are omitted for clarity.

Constructing a faithful in silico representation of a biological nanopore is the foundational step of any simulation study. The standard workflow (Figure 2a) begins with a high‐resolution crystallographic or cryo‐EM structure of the nanopore (here, *Staphylococcus aureus* α‐hemolysin, α‐HL). The protein is first aligned to have its transmembrane pore directed normal to the lipid bilayer and then embedded into a pre‐equilibrated lipid membrane patch. The size of the system patch, as well as the system's dimensions normal to the bilayer membrane, are typically chosen to avoid the interactions between the periodic images of the nanopore or the analyte. One should also be aware that the system's aspect ratio can affect the measured nanopore conductance [77, 78]. The atomic‐scale model of the nanopore is inserted into the atomic scale model of the membrane, the lipids overlapping with the nanopore are then removed. The resulting system is then solvated with explicit water and ionized to the desired molarity. Following that, the system is minimized and equilibrated while gradually releasing the positional and/or internal restraints to preserve integrity of the simulation system [79]. Following that, an external electric field is applied to produce the nanopore current of ions [37].

As illustrated in Figure 2b, the landscape of biological nanopores has diversified significantly beyond the archetypal α‐HL [68, 80]. While α‐HL remains a popular system for probing the physics of nanopore transport and exploring approaches to DNA or protein sequencing, other pores offer distinct sensing advantages. *Mycobacterium smegmatis* porin A (MspA) features a conical vestibule and a short, sharp constriction, providing enhanced spatial resolution for nucleotide identification [65, 66, 81], while CsgG has become the standard for high‐accuracy commercial sequencing [50, 82]. For small molecule and metabolite sensing, bacterial porins such as OmpF [62, 83, 84, 85] and FhuA [63, 64, 86, 87] are frequently employed; these pores provide highly specific internal chemistries ideal for studying antibiotic translocation and ligand‐binding kinetics. For proteomic applications, aerolysin has emerged as a superior sensor; its narrow transmembrane channel makes it exceptionally sensitive to the molecular volume, folding state and surface charge of peptides [31, 70, 88, 89, 90]. Wider, multi‐component pores offer a convenient platform for the study of larger analytes. Thus, intermediate‐sized toxins such as ClyA [72] and Fragaceatoxin C (FraC) [67, 91, 92] are often used to capture bulky macromolecular complexes that would clog narrower channels. Even larger pores, such as the α‐pore forming toxin YaxAB [75] and members of the MACPF/CDC family including PlyAB [74] and PLY [76], offer significantly wider apertures. Most notably, the recently introduced poly(C9) pore [73], with a diameter reaching tens of nanometers, represents a new class of “ultra‐wide” sensors. These large‐scale architectures are ideal candidates for the stochastic sensing of fully folded proteins and multi‐protein assemblies, providing a customizable platform for single‐molecule proteomics studies.

Transforming a static PDB structure of a biological nanopore into a physically meaningful MD trajectory requires careful system preparation. A critical first step is the assignment of protonation states. Standard physiological pH assumptions often fail inside the confined and highly charged environment of a nanopore. It is, therefore, recommended to check and assign the protonation states according to the results of local ${\mathrm{pK}}_{a}$ calculations, for example, using PROPKA [93] or H++ [94]. Another important consideration is system solvation. Simply overlaying a solvent box onto a membrane–protein complex can generate high‐energy steric clashes. Moreover, naive solvation may allow water molecules to penetrate and become trapped within the hydrophobic lipid tails, destabilizing the bilayer. Specialized equilibration protocols—often implemented through VMD plugins [95] or custom scripts—are therefore used to prevent solvent from entering the interface between the hydrophobic core of a lipid bilayer and the outer nanopore surface during early equilibration steps [96]. A common structural artifact in biological nanopore simulations is “ring piercing,” in which a lipid tail threads through an aromatic side chain or flexible peptide loop, leading to unphysical forces, structural distortions and an abnormal simulation termination. Using soft‐core potential minimization or careful initial packing procedures can reduce the likelihood of such artifacts. To avoid possible distortions of the protein nanopore structure, it is standard practice to apply harmonic positional restraints to the $C_{\alpha }$ atoms of the pore backbone, particularly within the transmembrane region. These restraints preserve the crystallographic geometry and provide a stable reference for open‐pore current calculations. Finally, to reduce computational cost, early nanopore MD studies often used truncated pore models (e.g., simulating only the membrane‐embedded region of MspA [97]). Although efficient, such truncations neglect entrance effects and electro‐osmotic flows generated by the extramembranous vestibule. Consequently, full‐length pore models are now preferred for achieving quantitatively accurate conductance and translocation behavior. Interested readers are directed to a recent study [78] that systematically examined the ability of the all‐atom MD method to quantitatively reproduce experimentally measured currents through biological nanopore MspA and its mutant variants.

### 3.2 Solid‐State Nanopores

Solid‐state nanopores refer to a highly versatile class of nanostructures produced either by bottom‐up chemical synthesis or top‐down nanofabrication [98]. While many types of artificial porous membranes have been developed for engineering applications, they are typically not suitable for ionic current sensing applications because the translocation signal from individual nanopores is overwhelmed by the background noise from all other nanopores. The first individual solid‐state nanopores were manufactured using the ion‐track method in polymer membranes [99] and, later, using either a focused ion or electron beam in thin silicon nitride (${\mathrm{SiN}}_{x}$) [100, 101] or silicon dioxide (${\mathrm{SiO}}_{2}$) [102] membranes. The introduction of dielectric breakdown methods [103] has democratized nanopore manufacturing at the cost of not knowing the nanopore dimensions precisely. The primary potential advantage of solid‐state nanopores over their biological counterparts lies in their mechanical and chemical stability and the ease with which their dimensions can be customized to accommodate a diverse range of analytes, from small molecules to large macromolecular assemblies [45]. Furthermore, nanopores in semiconductors [104] and some 2D materials, such as graphene [105, 106, 107] and molybdenum disulfide (${\mathrm{MoS}}_{2}$) [108], offer additional measurement modalities, for example, capacitance [109] or transverse conductance [110] sensing. However, practical application of solid‐state nanopores is plagued by multiple experimental challenges. While biological pores offer atomic‐scale reproducibility, solid‐state pores often suffer from fabrication‐induced variations in pore geometry and poorly defined surface chemistry. Nevertheless, the promise of seamless integration with semiconductor technology, of massive throughput and field deployment continues to fuel academic and industry research in this area. Interested readers are directed to reviews of solid‐state nanopore fabrication and applications [45, 111, 112].

A critical hurdle in the computational modeling of solid‐state nanopore systems is the forcefield disparity between biological and inorganic materials. While biological nanopores benefit from highly refined, “all‐in‐one” forcefields like CHARMM [113, 114, 115] or AMBER [116, 117, 118, 119] that accurately represent proteins, lipids, and nucleic acids in aqueous environments, solid‐state materials lack an equivalently transferable description. Instead, the materials science community relies on specialized potentials such as AIREBO [120, 121], Tersoff [122, 123], Stillinger‐Weber [124], and BKS [125], that are optimized for reproducing bulk mechanical, structural, and thermal properties of specific materials (e.g., graphene or silicon nitride). While these potentials accurately describe covalent networks within specific solid‐state materials, they were not developed to capture the behavior of these materials exposed to salty aqueous solutions nor their interactions with biomolecules, both essential for modeling nanopore translocation processes. Because bulk materials forcefields do not intrinsically define cross‐interactions with biomolecular species, hybrid parameterization strategies are often required. These typically involve constructing mixed Lennard–Jones parameters [126], assigning custom charges to the atoms at the surface and in the bulk of solid‐state membranes [127], and using grid‐based potentials to prevent irreversible adsorption of ions and other analytes onto the pore walls [128]. Below, we review the simulation protocols developed to address these challenges for each class of solid‐state nanopores.

Nanopores in silicon‐based materials, that is, silicon nitride (${\mathrm{SiN}}_{x}$) or silicon dioxide (${\mathrm{SiO}}_{2}$), were among the first used for single‐molecule sensing [101, 129, 130, 131]. A significant challenge in modeling these systems is ensuring that the inorganic surface interacts realistically with biomolecules and ions. To address this challenge, existing force‐fields describing solid‐state materials were transcribed in the language of biomolecular forcefields [126, 128] or developed from scratch to empirically match experimental material properties [127]. To build an all‐atom model of an experimental solid‐state nanopore system, there are two options. The simplest and the most commonly used one is to chisel out a nanopore from a crystalline silicon nitride (or silicon dioxide) membrane, add water and ions (Figure 3a). In doing so, one can preserve the accurate description of the membrane's internal mechanics and use the nominal partial charges of the inorganic atoms, along with the combinatorial rule for Lennard–Jones parameters, to describe the membrane's interactions with water, ions and biomolecules [126, 131, 132]. The drawback of this approach is that, depending on how the membrane is cut, one will impart a permanent dipole moment on the membrane because of its crystalline order. Furthermore, removal of individual atoms from their crystalline coordination at a membrane surface will create sites for near‐permanent binding of ions [133] and other charged moieties, such as the atoms of DNA backbone or charged groups of DNA bases responsible for Watson–Crick base pairing. The magnitude of such binding can be controlled by using a repulsive grid‐based potential acting on select molecular species [128, 134, 135].

**FIGURE 3:**
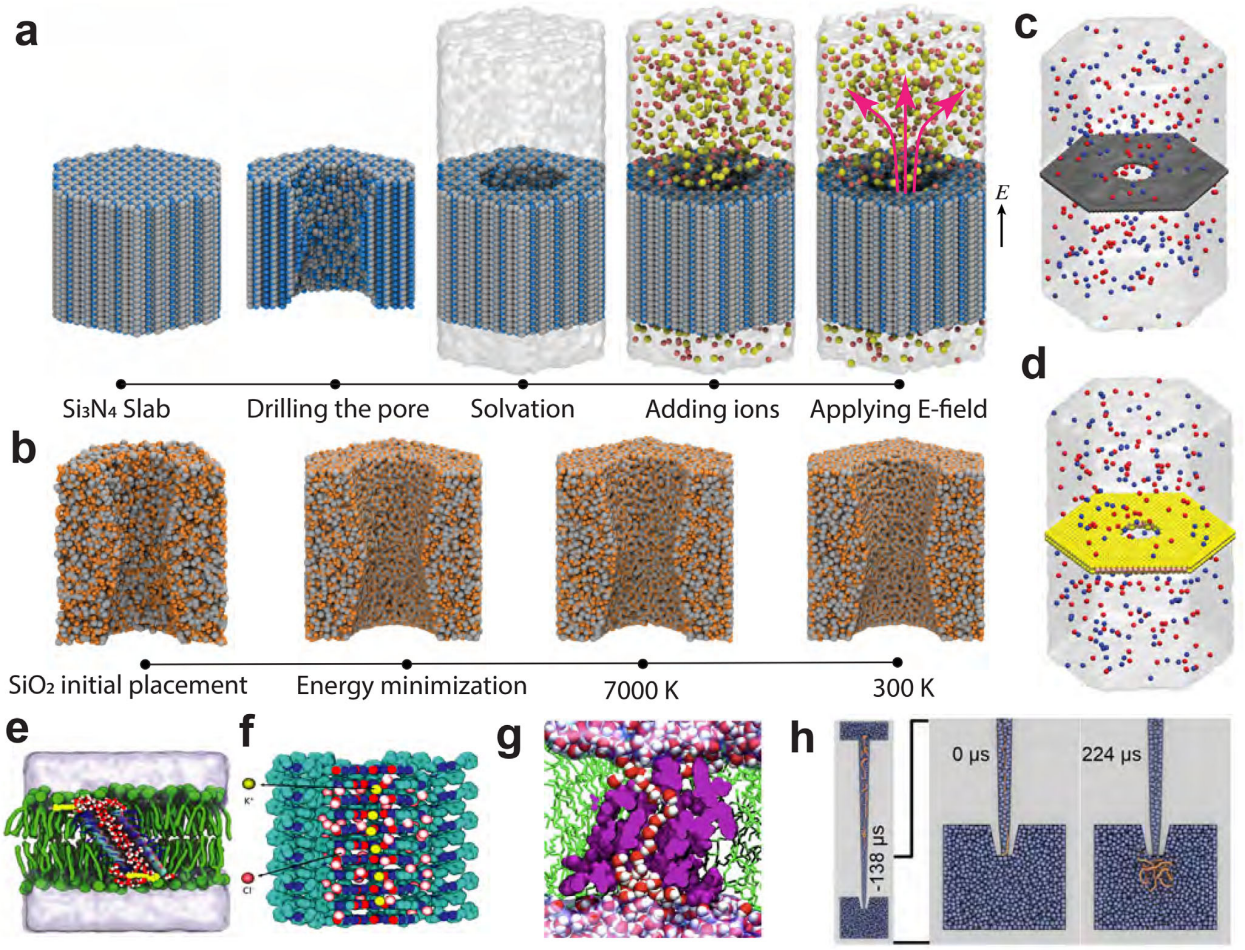
Solid‐state and synthetic nanopores. (a) Building an all‐atom model of a silicon nitride nanopore [79, 126, 136]. A nanopore is created by removing atoms from a crystalline membrane according to the desired geometry. Next, the membrane is submerged in a volume of electrolyte solution. After a short equilibration needed for the solvent to attain its equilibrium density, the system is simulated in a constant volume ensemble under external electric field to generate the nanopore ionic current. (b) Construction of a nanopore in amorphous SiO_2_ by thermal annealing [79, 137]. Pores in amorphous silicon dioxide (${\mathrm{SiO}}_{2}$) are formed through a specialized annealing process to ensure that the resulting surface has a realistic chemical structure. To achieve this, the system is initialized by defining the desired pore geometry—such as a conical or hourglass shape—using a 3D grid potential that acts as a “mold”. Silicon and oxygen atoms are initially placed randomly within the membrane region outside of this grid‐defined volume. A repulsive potential from the grid then forces the atoms to conform with the target geometry during a high‐temperature annealing phase, where the system is heated (typically to 6000–7000 K) and then slowly cooled. This process allows the atoms to relax into a stable, glassy state while maintaining a well‐defined pore surface. (c,d) Nanopores in 2D materials: graphene (panel c) and MoS_2_ (panel d). (e) Foldamer‐based synthetic nanopore embedded in a lipid bilayer membrane [138]. (f) Rosette nanotube porin formed by self‐assembly of guanine–cytosine rosette motifs [139]. (g) Synthetic peptide‐appended pillar[5]arene nanopore embedded in a lipid membrane [140]. (h) Grid‐based model of a glass nanopipette delivering genome‐size DNA into a dense protein solution [141]. Panel e is reproduced from the study by Roy et al. [138], Copyright 2021, *Springer Nature*, panel f from the study by Tripathi et al. [139], Copyright 2020, *American Chemical Society* panel g from the study by Shen et al. [140], Copyright 2015, *National Academy of Sciences* and panel h from the study byChau et al. [141], Copyright 2024, *Springer Nature* (under a CC‐BY 4.0 license), with permission.

A second option is to obtain a desirable model of an oxidized silicon surface through a thermal annealing simulation (Figure 3b) [127]. In such simulations, an initially crystalline or amorphous model of the membrane is melted at high temperature and then cooled down, while an external grid‐force potential maintains the nanopore shape [137]. Depending on the parameters of this annealing procedure, the configuration of the surface atoms can be fine‐tuned to have the desired number of the exposed silanol groups, which in turn controls the overall hydrophobicity of the simulated surface [127]. Upon completion of the annealing procedure, atoms of the membrane are positionally restrained using the spring constant and partial charge combination determined to match the experimental dielectric response of the material. By setting the average surface hydrophobicity and the membrane material's dielectric constant, this approach can model a variety of nanopore systems [142, 143, 144].

Two‐dimensional (2D) nanopores, fabricated from single‐atom‐thick sheets such as graphene [105, 106, 107, 145] or molybdenum disulfide (${\mathrm{MoS}}_{2}$) [108, 146] represent the ultimate limit in sensor miniaturization. These materials are highly valued for their exceptional mechanical strength, high electrical conductivity, and low surface friction [147, 148, 149]. In the context of DNA sequencing, graphene and ${\mathrm{MoS}}_{2}$ offer complementary strengths: ${\mathrm{MoS}}_{2}$ nanopores typically exhibit lower noise in the low‐frequency regime with a higher ionic current blockade amplitude, while graphene interacts more strongly with DNA, resulting in a longer, more resolvable translocations [150, 151]. To address the forcefield requirements of these materials, the INTERFACE forcefield [152, 153] is a robust option, as it includes parameters for both carbon‐based and ${\mathrm{MoS}}_{2}$ materials. A significant advantage of this framework is that it supports the functionalization of atoms, typically with hydrogen, at the pore edge to better mimic experimental surface chemistry. The process of utilizing this forcefield and constructing the simulation system is streamlined through the CHARMM‐GUI platform [154, 155], which automates the setup of complex bio‐inorganic interfaces. Alternatively, one can employ the same structural modeling protocol used for silicon nitride (Figure 3a) to construct the nanopore geometry by removing atoms from a 2D sheet. It is important to stress, however, that care must be taken when choosing an existing forcefield for MD study of a 2D materials interacting with biomolecules as different parameterizations of the same material can lead to drastically different simulation outcomes [156]. Typical all‐atom models of graphene and ${\mathrm{MoS}}_{2}$ nanopores are shown in Figure 3c and Figure 3d, respectively. Models of such materials often lack optimized angle and dihedral angle potential parameters, and therefore it is usually necessary to apply harmonic restraints to the membrane atoms to prevent large, unphysical deformations of the thin sheet.

Supramolecular chemistry offers an alternative route to producing structurally defined synthetic nanopores [3, 157, 158]. One prominent strategy involves the use of foldamers, synthetic polymers engineered to adopt well‐defined helical or tubular secondary structures, which can be designed to form biomimetic water channels (Figure 3e) [138, 159]. Foldamers provide exceptional control over the channel interior, enabling precise alternation of hydrophobic and hydrophilic monomers to promote rapid water transport while maintaining high ionic selectivity [138, 160]. Another robust approach exploits self‐assembling supramolecular motifs, such as rosette nanotubes formed through hydrogen bonding between guanine–cytosine building blocks (Figure 3f) [161]. These assemblies mimic the structural organization of biological porins while offering enhanced chemical stability and modular functionalization for sensing applications [139]. Alternatively, macrocyclic scaffolds, including pillar[n]arenes, serve as rigid, predefined apertures that can be functionalized with hydrophobic “anchors” to facilitate insertion into lipid membranes (Figure 3g) [162]. Peptide‐appended pillar[5]arene unimolecular channels, for example, have demonstrated exceptionally fast water transport rates comparable to those of biological aquaporins [140, 163]. Modeling such nanopore systems begins with defining a unit building block using crystallographic or other structural data, and then using a general forcefield (CGenFF) webserver [164] to derive forcefield parameters compatible with the CHARMM model. For lipid‐embedded nanochannels, a lipid bilayer is pre‐equilibrated before insertion of the nanochannel [165, 166]. Following that, the pore is equilibrated under constant pressure and temperature conditions while harmonically restraining all nonhydrogen atoms of the pore.

Solid‐state nanopores can reach sizes on the order of 100 nm in length and diameter, which are too large to simulate with an all‐atom MD approach. For such systems, we can employ coarse‐grained (CG) models, which can provide orders of magnitude speed‐ups, but represent the system with reduced resolution and accuracy [167]. In CG simulations of nanopore systems, we typically use implicit solvent and Debye‐Hückel electrostatics to represent charge–charge interactions and use an external electrostatics solver to determine the three‐dimensional electrostatic potential in the pore. We commonly employ COMSOL [168] to compute the effective potential as it simplifies the development of a model that additionally incorporates Navier‐Stokes flow [167]. Additionally, a steric potential must be included to prevent the analyte from entering the membrane and pore walls. After the nanopore potentials are assembled, we construct a model of the analyte using a previously developed CG model, such as mrDNA [169], a 2‐site‐per‐nucleotide model of single‐stranded DNA (ssDNA) [170], or any of several peptide models [171, 172, 173, 174]. Because these models lack explicit solvent, it is essential to estimate the effective electrophoretic force experienced by the analyte molecule in the electrolyte of interest, which, due to the motion of counterions and the associated solvent flow, can be significantly lower than the electric force computed directly from the product of local field and charge. For example, conducting an all‐atom simulation of a harmonically restrained analyte under applied electric field can measure the effective electrophoretic charge of the analyte [175]. Finally, the simulation can be performed using either Brownian or molecular dynamics, allowing the motion of the analytes through the nanopores to be observed, for example DNA translocation through a glass nanopipette filled with a dense protein solution [141] (Figure 3h).

### 3.3 DNA Origami Nanopores

DNA origami refers to a method of folding a long DNA “scaffold” strand, typically taken from a viral genome, into a designed nanoscale shape using many short, synthetic “staple” strands in a one‐pot reaction [176]. Functional decorations to an origami object can be placed with sub‐nanometer precision because the base pairing property of DNA yields highly predictable molecular positioning. By strategically attaching hydrophobic chemical groups (including cholesterol, alkyl chains, and porphyrin) to a DNA origami object, it is possible to induce the DNA object to bind and even insert into a lipid bilayer membrane [177, 178]. Such insertion must overcome the significant energetic cost of bringing a highly charged DNA object proximal to the low dielectric, counterion‐free environment of the membrane. Because a DNA origami structure can be decorated with a variety of chemical groups, this approach holds promise for the creation of custom functional nanopores [179]. By computationally mimicking the experimental steps of origami construction, decoration with hydrophobic moieties, insertion into a membrane and solvation with water and ions, all‐atom MD simulations can be used to study such nanostructures [180, 181, 182], accelerating the design process (Figure 4a). Extensive reviews on the fabrication and applications of DNA nanopores are available for interested readers [44, 183].

**FIGURE 4:**
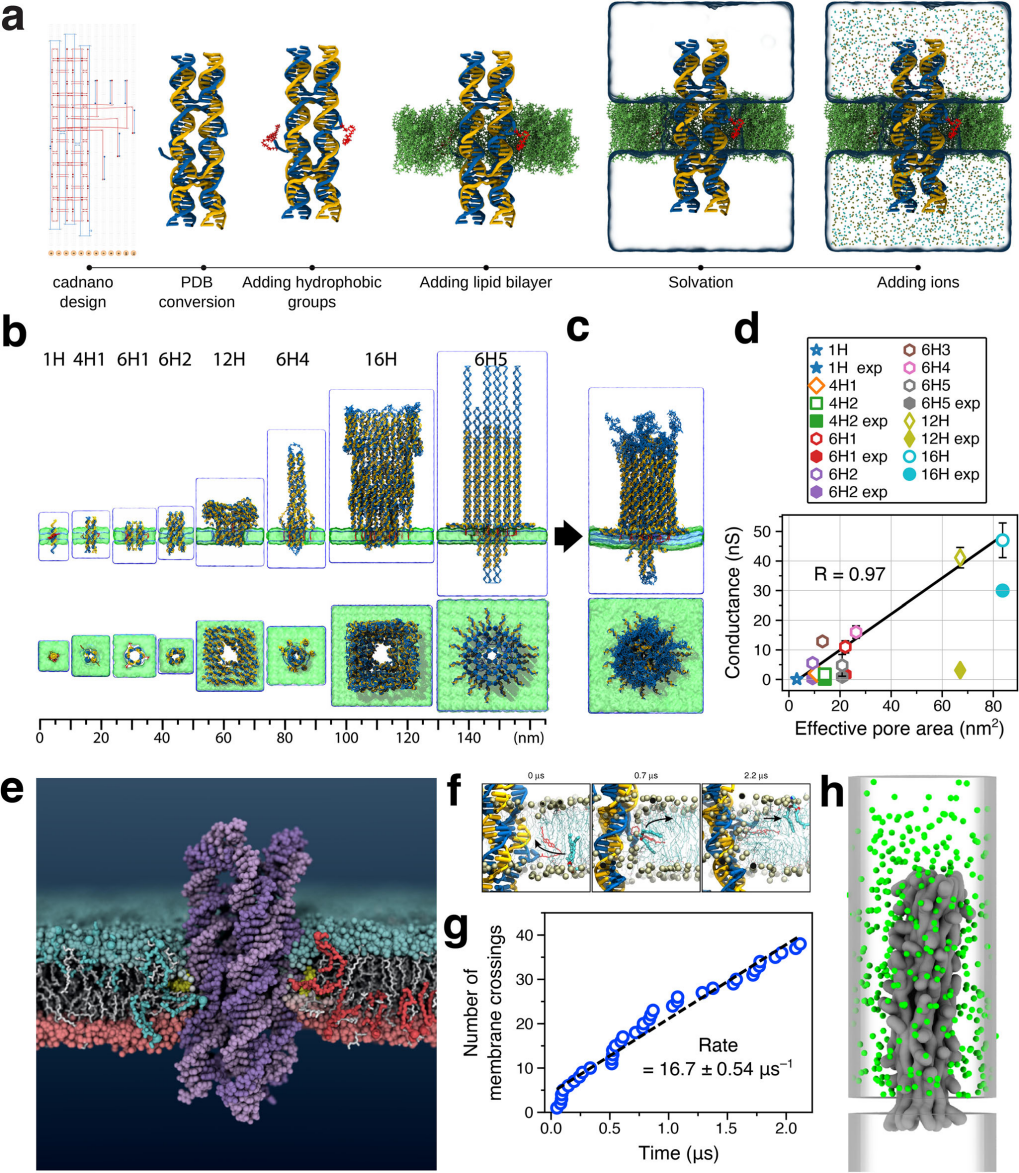
DNA origami nanopores. (a) Procedures for generating an all‐atom MD model of a DNA nanopore system. Most DNA nanostructures are designed using the caDNAno program [184], and can be converted to a fully atomistic representation using, for example, the mrDNA structure prediction [185] or the cadnano2pdb [186] web service. The atomistic model is then decorated with hydrophobic groups before insertion into a bilayer and solvation with a pre‐equilibrated volume of electrolyte [187]. (b) Initial atomistic models of DNA nanopores arranged from smallest to largest, shown from the side (top row) and from above (bottom row). DNA nanopores (blue and yellow) decorated with hydrophobic molecules (red) are embedded in DPhPC lipid bilayers (green) and submerged in solvent (blue semitransparent surface). (c) Final configuration of the largest 6H5 nanopore [177] after 100 ns all‐atom MD simulation. (d) Ionic conductance dependence on pore area for the DNA nanopores shown in panel b. The ionic conductances were averaged over simulations at ±30 and ±100 mV, except for 6H2 and 6H3 which were simulated at 50, 100, 200, 400 and 600 mV [180]. The effective pore area was estimated from the number of water molecules in the 1‐nm slab centered on and aligned with the bilayer. Experimentally measured conductances are shown, if available, as filled symbols. (e,f) Lipid scrambling activity of the 4H1 system. Scrambling of lipids occurs when lipids diffuse across the toroidal pore formed by the DNA nanopore. (g) Number of lipid crossing events observed in an all‐atom MD simulation of the 4H1 nanopore [188]. (h) Coarse‐grained model of TAMRA dye molecules surrounding a DNA nanopore embedded in an implicitly‐represented lipid bilayer membrane allowed characterization of the DNA nanopore permeability to the dye molecules [189]. Panels b–d were reproduced from the study by Li [190]. The 1H and 16H systems in panel b were adapted from the study by Göpfrich et al. [181, 182]. Panels f‐g were adapted from the study by Ohmann et al. [188], Copyright 2018, *Springer Nature* under a CC‐BY 4.0 license and panel h from the study by Li et al. [189], Copyright 2022, *American Association for the Advancement of Science*.

All‐atom or even coarse‐grained simulations of DNA origami folding remain computationally intractable. Hence, one begins computational modeling of a DNA origami nanopore assuming the design folds, which experimentally can be verified at the level of a gel electrophoresis measurement. Initial structural relaxation of a large nanopore from its idealized folded configuration can be simulated using either implicit solvent [191] or coarse‐grained [169] models, but reproducing subtle balance of forces at the DNA–lipid membrane interface typically requires an all‐atom MD approach [180]. When a DNA duplex is decorated with enough hydrophobic groups, the hydrophobic tails of the lipid membrane can avoid the DNA backbone by forming a toroidal pore surrounding the duplex [181]. A similar principle can be used to build larger nanopores [182, 188, 192] (Figure 4b). Over the past ten years, we have simulated a number of DNA nanopores using the all‐atom MD method, ranging in size from a single duplex [181] to a large multi‐helix assembly (Figure 4c). Our simulations show that the simulated ionic current through a DNA origami nanopore is roughly proportional to the nanopore's cross‐sectional area within the plane of the lipid bilayer membrane (Figure 4d).

Microseconds all‐atom MD simulations of a DNA origami nanopore revealed that lipid molecules are able to move from one leaflet of the bilayer to the other, through the toroidal nanopore by diffusion [188] (Figure 4e,f). The rate of such inter‐leaflet transport was found to exceed, by many orders of magnitude, the rate of lipid scrambling facilitated by biological enzymes— scrambles [188] (Figure 4g). This computationally predicted behavior was corroborated by experiments performed by the Keyser lab [188, 193].

The large size of some DNA origami nanopores requires multi‐resolution computational approaches for the computational investigation of their properties. For example, the Schulman lab designed DNA origami nanotubes to conduct dye molecules over a length scale of hundreds of nanometers between lipid vesicles [189]. To study the translocation of dyes through these nanotubes, we applied our coarse‐grained mrDNA model to the DNA nanostructure using spherical beads to represent the dye molecules and a 3D grid potential to represent the lipid bilayer (Figure 4h). The model allowed characterization of the permeability of the nanotube walls to the dye molecules as a function of their size [189].

## 4 Simulation Parameters

All‐atom MD discretizes Newton's equations of motion for a classical description of atoms that interact through a set of prescribed interactions, interleaving force evaluations with integration that advances the coordinates and momenta by a small unit of time known as the timestep [194, 195]. Hence, even the simplest MD simulations of a nanopore will involve dozens of parameters and many choices. Below, we briefly review the algorithms, parameters, and our preferred practices for all‐atom MD simulations, emphasizing special considerations for nanopores.

One of the most critical decisions in designing an all‐atom MD simulation is the choice of a molecular forcefield, which refers to a set of potentials and the associated parameters that underpin the evaluation of interparticle forces. Currently, there are two major families of biomolecular forcefields, CHARMM [113, 114, 115] and AMBER [116, 117, 118, 119], that have been developed and revised over many decades. The forcefields have many similarities but notably distinct approaches to parametrization, though both generally employ a mixture of experimental vibrational spectra and quantum calculations to inform their parameters. In general, both forcefields represent biomolecular systems well and are capable of reproducing many experimental results, including the stability of crystallographic structures, qualitative trends in hydration free energies [196], and even the temperature dependence of folding for select small proteins [197], while not being specifically parameterized to do so. We have typically used the latest‐available CHARMM forcefield, which was historically developed for protein simulations. However, both forcefields approximate atomic interactions and therefore contain artifacts. For example, we have shown that both forcefields overestimate the interaction free energy between a variety of oppositely charged molecular groups, including ion–nucleic acid [198, 199], peptide–nucleic acid, peptide–peptide and nucleic acid–lipid interactions [200, 201] that are often abundant in nanopore simulations. These particular shortcomings were addressed by introducing the CUFIX corrections for both forcefields [198, 199, 200, 201], which surgically replace the Lennard‐Jones parameters for select atom pairs with values tuned to reproduce experimentally‐measured osmotic pressure data for binary solutions of model compounds [202, 203].

Water comprises over 80% of a typical simulation volume. Both CHARMM and AMBER forcefields were originally parameterized using TIP3P [204], a three‐point model of water that offers decent representation of water properties but underestimates the water viscosity by a factor of 2.5. The TIP3P model is used widely by the community and in our own work because of its low computational cost and compatibility with the most commonly used biomolecular forcefields. The more complex TIP4P‐D water model [205] includes an extra dummy particle on each molecule that improves the description of London dispersion forces, resulting in better representation of disordered peptides and also a more realistic viscosity [206]. While we typically use CHARMM forcefields, we previously found [207] that intrinsically disordered peptides are best represented by the AMBER ff99${\mathrm{SB}}^{*}$‐ILDN variant [208, 209, 210] combined with the TIP4P‐D water model, and that the majority of other forcefield choices promote strongly collapsed peptide configurations, inconsistent with experimental data. When it comes to modeling blockade currents from DNA strands, using the latest generation of forcefields does not guarantee an improved realism [78] as the forcefields are not typically refined using experimental nanopore translocation data. In addition to polarizable water, polarizable variants of the CHARMM [211] and AMBER [212, 213] forcefields have been under development for some time, but these models introduce even more parameters and are still being critically evaluated, with some properties being better captured by their non‐polarizable counterparts [214, 215, 216]. This, combined with a higher computational cost, has so far limited the widespread adoption of polarizable models for MD simulations of nanopore transport.

Aside from the choice of a forcefield, most algorithms and parameter choices in an MD simulation involve a trade‐off between accuracy and performance. The most expensive computation during an MD step is the evaluation of nonbonded (van der Waals and electrostatic) interactions. Direct evaluation of these forces is extremely costly and is limited in practice to a cutoff distance of 10–13 Å, but is supplemented by a Fourier space evaluation of the long‐range forces using the particle mesh Ewald (PME) method [217]. The PME method requires that the system is electrostatically neutral and employs periodic boundary conditions, though neither requirement is particularly problematic in practice. Electrostatic neutrality arises naturally for charged objects when sufficiently padded by electrolyte solution, whereas using periodic boundary conditions avoids the introduction of artifacts at the edge of a system and can facilitate a steady state flux, which is particularly useful in simulations of nanopores [218].

Once all the forces for a given configuration have been evaluated, integration of Newton's equations can be performed to advance the system's coordinates and momenta. The timestep used to advance the system coordinates is, in general, limited by vibrations of the bond between a hydrogen atom and its parent atom, which has a typical period of ∼10 fs. Constraining bonds involving hydrogen atoms using the SHAKE [219] and RATTLE [220] algorithms has allowed for using a 2 fs time step. Further efficiency is achieved by evaluating slowly‐changing long‐ranged electrostatic forces only every other step with “multiple timestepping”. Even larger timesteps, up to 4 fs, can be taken by employing hydrogen mass repartitioning (HMR) [221, 222], whereby some of the mass of each atom is redistributed to its bound hydrogen atoms. While HMR has been shown to preserve structural properties of membrane systems such as area‐per‐lipid and electron density profiles, it can affect kinetic properties such as diffusion constants [222]. A test simulation comparing results using 2 and 4 fs timesteps is therefore recommended before performing production simulations.

A thermostat is generally employed during nanopore simulations to avoid issues related to small drifts in total energy during long simulations and to keep the temperature constant when externally applied forces inject energy into the system, such as that of an applied electric field or pressure gradient. The most commonly used is the Langevin thermostat [223], where each atom is subject to a drag force proportional to its velocity and random forces that together satisfy the fluctuation–dissipation theorem. We recommend using a 0.1–1 ${\mathrm{ps}}^{-1}$ damping coefficient to minimally perturb the dynamics of the system. However, when a steady‐state flux is present, a momentum‐conserving thermostat, such as Lowe‐Andersen [224], is preferred. Alternatively, the Langevin thermostat can be applied with a larger damping coefficient just to the atoms of the membrane to avoid applying an artificial drag force counteracting the steady state flux.

Approximations employed during an MD simulation, primarily the discretization of the equations of motion [225], numerical artifacts of the PME calculation [226], and the action of the thermostat can result in a drift of the system's center of mass. Most MD simulation programs can optionally remove the total momentum from a simulation system at each timestep. Such behavior can be beneficial for accurate determination of effective forces [227, 228], but can otherwise lead to serious artifacts for example, suppression of nanopore flux. Hence, it is important to familiarize oneself with the detailed behavior of their chosen simulation program and examine the consequences on turning specific flags on and off.

## 5 Modeling Transport of Analytes

Following the assembly, a nanopore system typically undergoes an equilibration phase to ensure gradual structural relaxation into a physically valid configuration. While these equilibration simulations can characterize the distribution of ions and solvent, the formation of electrical double layers, as well as analyte–nanopore interactions, the functional utility of a nanopore sensor is fundamentally defined by its response to external electric field [5, 68]. To recapitulate this experimental situation, nanopore systems are simulated under external electric field, using a thermostat to dissipate heat and maintain the system's thermal equilibrium. However, because the capture of an analyte by the nanopore is a stochastic process, MD simulations of analyte transport rarely begin with having the analyte randomly placed in the simulation unit cell. Furthermore, because the experimental translocation timescale typically exceeds the microsecond timescale available to brute‐force MD, an observation of a complete translocation often requires methods to speed up the translocation process in the simulation.

One obvious method to solve the analyte capture problem is to place the analyte near [126] or half‐way through [229] the nanopore (Figure 5a). While doing so does not permit computational determination of the capture rate, this approach guarantees observation of a translocation process if the force driving the analyte transport is sufficiently strong. The most commonly used method to accelerate simulation of analyte nanopore transport is to apply an electric field of a magnitude much higher than the experimental one. In an MD simulation, the experimental transmembrane bias is reproduced by the application of a uniform electric field ($\vec{E}$) normal to the membrane. The field exerts a nominal force on each and every charged atom in the simulation systems, including water and ions, which rearranges their microscopic configuration. The combined actions of the applied electric field and of the reaction field from the rearranged atoms produce a highly non‐uniform distribution of the electric potential (Figure 5b) with nearly uniform regions away from the membrane and a sharp variation in the nanopore region [37]. Importantly, when an analyte enters the nanopore, it further perturbs the local charge distribution and electrostatic environment, and this dielectric heterogeneity is fully captured by the all‐atom MD framework without any additional assumptions, as the response of water molecules and ions to the presence of the analyte is treated explicitly at atomic resolution. The desired transmembrane voltage (V) can be “dialed in” by choosing the magnitude of the applied electric field according to the following formula: $E=-V/L_{z}$, where $L_{z}$ is the length of the simulation unit cell along the direction of the applied field [37, 230, 231]. Early MD simulations of nanopore transport [126, 128, 131, 132, 232, 233] required artificially high electric fields to observe translocation events within tens of nanoseconds. Using such high field is no longer necessary as other methods have been developed to accelerate simulations of analyte transport.

**FIGURE 5:**
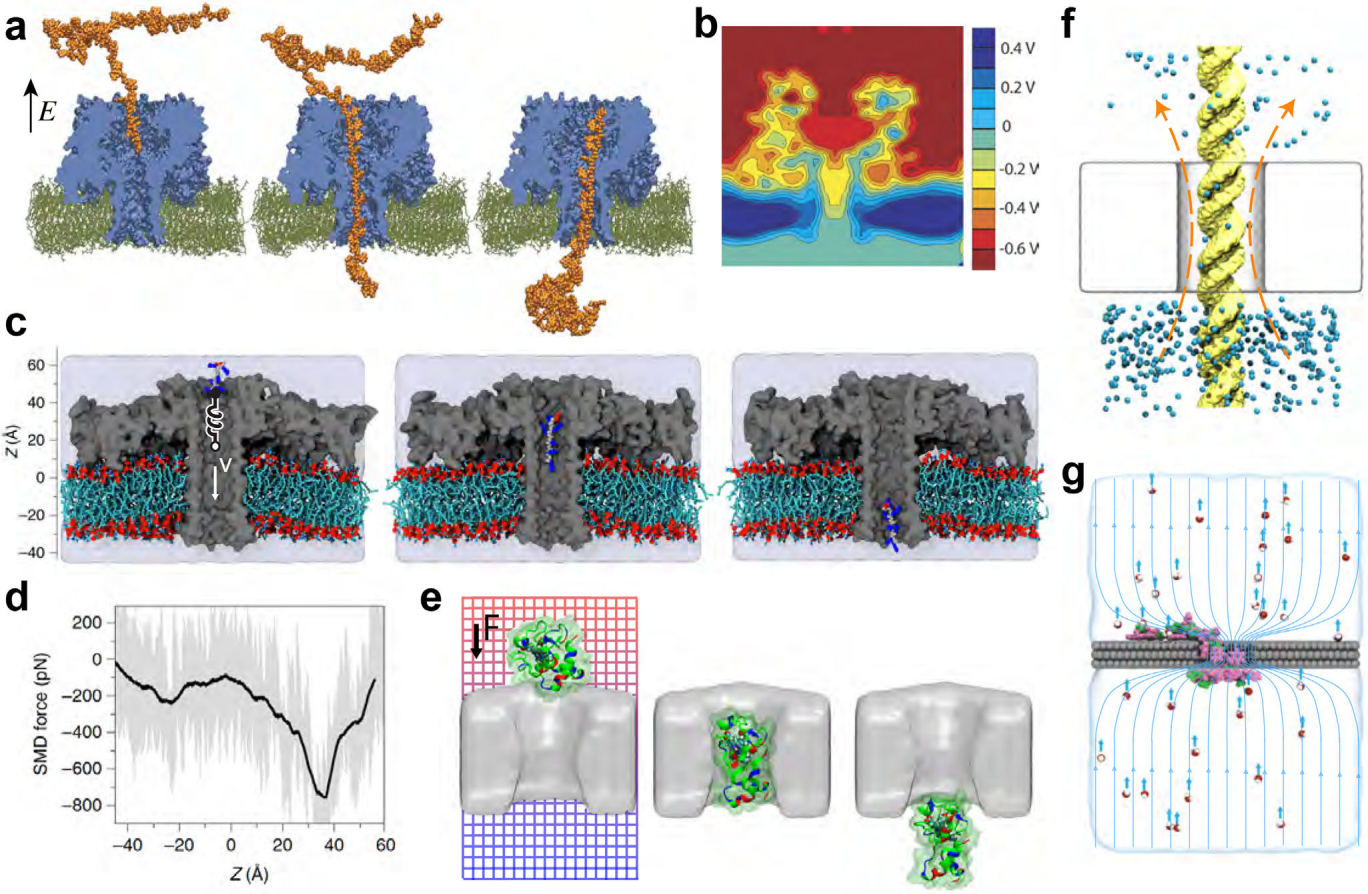
MD simulations of analyte transport through nanopores. (a) MD simulations of a DNA strand translocation through an α‐HL nanopore. A constant external electric field is applied across the membrane to generate an electrophoretic force that drives translocation of the DNA strand [136]. (b) Steady‐state distribution of the electrostatic potential in and around α‐HL when a constant electric field is applied to produce a 600 mV transmembrane bias [37]. (c) Steered MD simulation of a polyarginine peptide (blue spheres) translocation through the transmembrane pore of aerolysin (gray) [89]. A template particle (black) is moved with a constant velocity, pulling the analyte through the nanopore by coupling the template' and analyte's coordinates via a harmonic spring potential. (d) The force exerted on the peptide during the SMD simulation [89] featured in panel c. The instantaneous SMD force is shown in gray and its 5 Å running average in black. (e) Translocation of cytochrome c through a SiN nanopore driven by Grid‐SMD [234]. The biasing potential, schematically represented by a color‐coded grid, is applied to guide the analyte along the pore axis. (f) Transport induced by a concentration gradient. A potential gradient is imposed at the simulation boundaries, creating a diffusive flux that promotes analyte passage through the nanopore. (g) Pressure‐driven flow generated by applying a constant external force to individual water molecules in the system [12]. The force produces a hydrodynamic flow field that drives the analyte through the nanopore, mimicking the action of a pressure differential between the two reservoirs. Panels a and b are adapted from the study by Aksimentiev [136], Copyright 2010, *Royal Society of Chemistry*, panels c and d from the study by Ouldali et al. [89], Copyright 2020 *Springer Nature*, panel e from the study by Tripathi et al. [234], Copyright 2021 *National Academy of Sciences*, and panel g from the study by Wilson et al. [12], Copyright 2016, *American Chemical Society*.

The choice of applied voltage in an MD simulation of a nanopore system involves several practical considerations. For exploratory simulations, the applied voltage can be chosen higher than experimental one to accelerate the transport and make simulations computationally feasible. In practice, the voltage range for MD simulations of biological nanopores is limited to 0.01–1.2 V and the higher voltages produced electroporation of a lipid bilayer, unless it is additionally restrained. Solid‐state nanopore can withstand much higher fields, up to 10 V, depending on parameters of the system. Second, excessively high electric fields can cause atoms to move beyond the maximum displacement thresholds enforced by MD engines, leading to numerical instabilities and simulation crashes. Combining a high electric field with a high damping coefficient of the Langevin thermostat can considerably improve the stability of ahigh voltage MD run. Third, while high voltages in experimental settings can cause water electrolysis, it is not a concern for a classical MD simulation as the non‐reactive forcefields do not allow bond breaking or formation. Finally, high electric fields can compromise the structural integrity of both the nanopore and the lipid membrane. For biological nanopores, applying harmonic restraints on the protein backbone combined with a network of elastic restraints on the non‐hydrogen atoms of the nanopore and the surrounding lipids is recommended to prevent deformation and membrane rupture. For solid‐state nanopores, harmonic restraints on the nanopore atoms are similarly advised to maintain structural stability under high applied fields.

While scaling up the electric field can be useful for qualitative assessment of the translocation behavior, such simulations can produce distortions in the conformations of the translocating molecules and disrupt the integrity of a lipid bilayer membrane. Steered molecular dynamics (SMD) [235] is a convenient simulation protocol that guarantees the observation of nanopore capture and translocation. In this method, a harmonic spring is attached between the center of mass of an analyte (or a specific group of atoms) and a dummy atom (Figure 5c). By pulling this dummy atom along a predefined axis at a constant velocity, researchers can guide the analyte through the pore within a timescale defined by the pulling speed. SMD is particularly valuable for “scanning” a pore to systematically compute blockade currents [89], identifying translocation barriers (Figure 5d), assessing local friction [236] and measuring the effective forces on the translocating molecules [175]. Furthermore, SMD enables the study of structural transitions, such as the unfolding of peptide fragments [237] or the unzipping of nucleic‐acid hairpins [232], necessary for threading analytes through nanopore constrictions. In contrast to a constant force simulation, the SMD method guarantees nanopore translocation, often at a price of applying unphysically large forces to the analyte. When using SMD for nanopore transport simulations, the choice of the target atom group is critical. For example, using the center of mass as an SMD target for a flexible polymer will not guarantee nanopore entry, whereas pulling on one of its ends by high force can produce unphysical structural distortions [238]. Another key consideration is the spring constant of the harmonic restrain used to pull the analyte. A high spring constant ensures the analyte strictly follows the dummy atom but results in significant force fluctuations, whereas a small constant allows for more natural responses to local potential barriers at the cost of precision. A practical rule of thumb is to select a spring constant that is as loose as possible (to reduce fluctuations in the applied force) while remaining stiff enough to ensure that expected forces do not cause a displacement exceeding the desired spatial control over the translocation trajectory.

Grid‐steered molecular dynamics (G‐SMD) [238] offers a more experiment‐faithful approach to driving transport compared to standard SMD by utilizing a precomputed three‐dimensional potential, obtained either from an open‐pore MD simulations (Figure 5b) or using a continuum electrostatics solver, to drive the translocation process. Unlike traditional SMD, which typically couples a harmonic spring to the center of mass of an analyte, G‐SMD applies an arbitrary external potential defined on a grid directly to desired target atoms (Figure 5e). This allows the force to be position‐dependent for each individual atom and account for the atom's electrical charge. Importantly, the coupling between the applied grid potential and the force experienced by the atoms can be selectively amplified, allowing for the application of a high effective bias without disrupting integrity of the simulation system. As a result, the translocation process is accelerated by many orders of magnitude without introducing high‐bias artifacts. The G‐SMD method has enabled systematic study of voltage‐driven capture and unfolding of proteins [234, 239], and translocation of ssDNA [238] and proteins [135] through various nanopore architectures.

When electric field‐driven effects are undesirable or the transported species is neutral or weakly charged, transport can be driven by a concentration gradient (Figure 5f). In this regime, molecules move down a chemical potential gradient by diffusion, with the resulting flux governed by Fick's law. Experimentally, this is implemented by establishing a solute concentration difference across a membrane‐spanning nanochannel. Conceptually, concentration gradient‐driven transport can be simulated by initializing the system having unequal solute concentrations on the opposite sides of the membrane and allowing the system to relax diffusively [240]. In practice, however, the use of periodic boundary conditions typically leads to quick dissipation of the concentration gradient without substantial nanopore transport. While using a grand‐canonical MD protocol [241] can maintain the desired concentration gradient, such simulations are typically too slow to be practical. Using a double membrane system to define compartments of high and low ion concentration [242, 243] eliminates the periodic boundary condition problem, but requires high solute concentration to maintain the concentration gradient. A convenient alternative is to apply an external potential to select solute species and thereby locally increase their concentration, effectively realizing a Neumann‐type boundary condition [244]. Alternatively, a mechanical barrier, such as a graphene sheet, can be positioned at the boundary of the simulation box [245]. While using such a barrier will prevent atoms from crossing the periodic boundary of the system, the barrier itself can introduce simulation artifacts such interfacial water polarization.

Another alternative to electric field‐driven transport is the application of a pressure gradient, which is particularly effective for the translocation of neutral or weakly charged analytes. In an MD simulation, this can be implemented by applying a constant body force to each water molecule [246], producing a uniform flow equivalent to that generated by a hydrostatic pressure difference across the membrane (Figure 5g). The resulting steady flow of water carries solutes through the nanopore regardless of their charge, enabling controlled translocation even when electrophoretic driving is ineffective or undesirable [12]. The pressure gradient method provides a purely mechanical means of transport control, in which water flow, rather than electric field, acts as the primary driver of molecular motion, and is closely related to electro‐osmotic transport regimes where ion‐driven water flow plays an analogous role [237, 247].

While all the above approaches accelerate analyte translocation, the choice of method carries distinct trade‐offs between physical realism and computational efficiency. It is worth noting that electric field, concentration gradient, and hydrostatic pressure‐driven transport all have direct experimental counterparts, making simulations employing these methods more readily comparable to experimental observations. SMD and G‐SMD, on the other hand, are purely computational approaches and therefore are best suited for exploring translocation pathways and generating a conformational ensemble rather than to making quantitative estimates of the kinetic quantities. High electric fields are the simplest approach but can distort molecular conformations at excessive voltages. SMD guarantees translocation and is ideal for scanning blockade current dependence on the analyte positions [89] (when combined with SEM [248]), but can impose unphysical forces on flexible analytes. G‐SMD applies a position‐dependent potential to individual atoms, enabling accelerated translocation with better conformational fidelity, making it preferable when structural accuracy is critical. Concentration gradients and hydrostatic pressure simulations are best suited to model transport of neutral or weakly charged analytes, when the electrophoretic forces are ineffective. The choice of the simulation method therefore depends on the specific experimental situation and the available computational resources.

## 6 Active Nanopores

While most nanopores passively conduct ions, water and analyte molecules down the electric field gradient, some nanopores can be engineered to exercise further control over the translocation process or enable additional sensing modality. Thus, nanopores in multilayer doped silicon–insulator–doped silicon membranes have been theoretically introduced as sensors of a DNA's electric field [104, 249, 250]. Being a good electrical conductor, a graphene membrane can itself be electrically biased relative to the solution. Graphene charging was theoretically shown to regulate the speed of nanopore ssDNA transport and produce conformational transitions in the structure of adhered ssDNA molecules [251]. Adding graphene to both sides of an insulating membrane creates a nanopore capacitor (Figure 6a). MD simulations have shown that the magnitude and selectivity of ionic current through a nanopore in such a membrane can be precisely regulated by the charge state of the capacitor's plates (Figure 6b), which also provides a means to control the electro‐osmotic flow [252].

**FIGURE 6:**
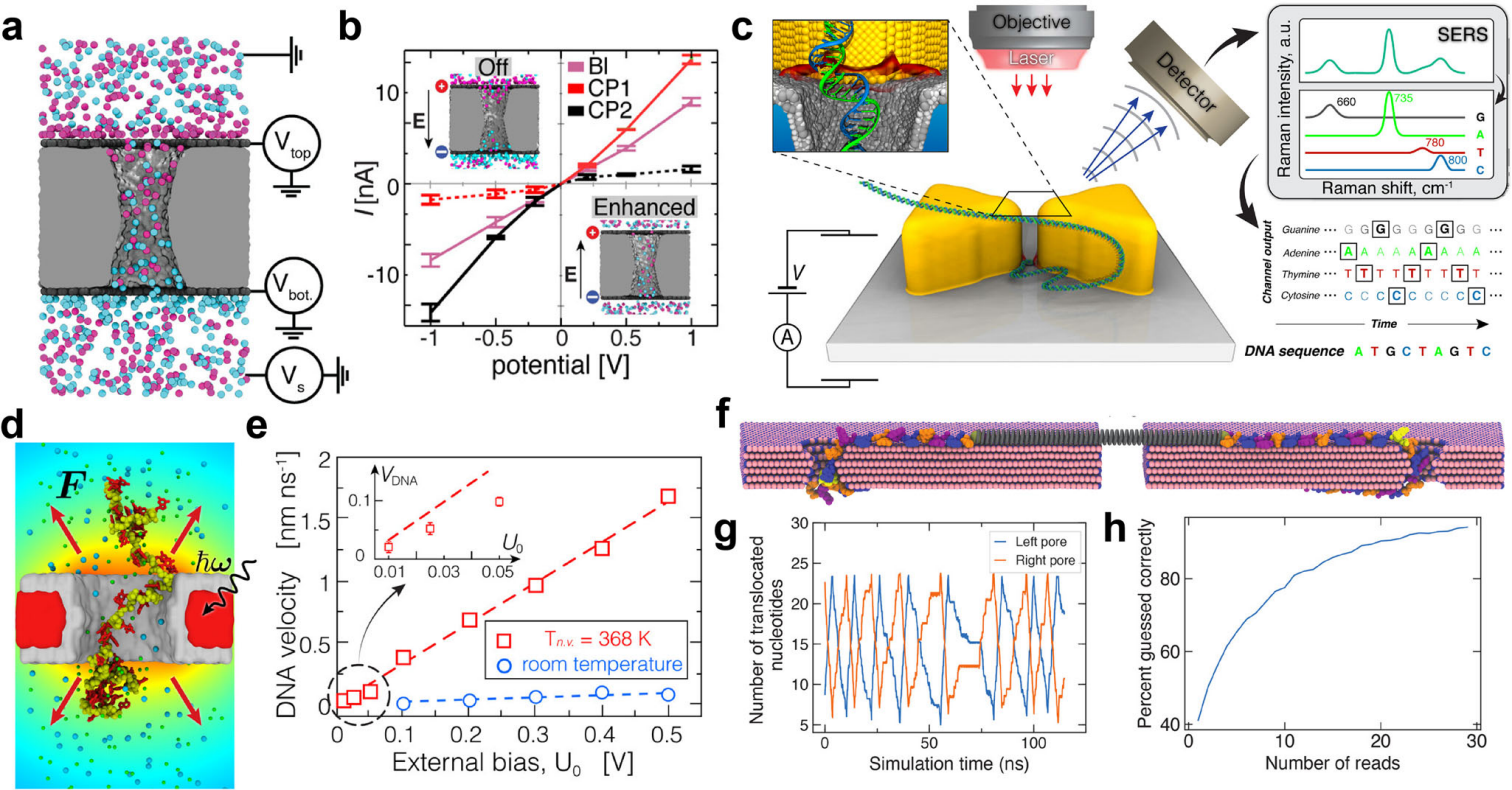
MD simulations of active nanopore systems. (a,b) Atomic model (a) and current‐voltage characteristics (b) of a graphene nanopore capacitor allows for regulation of current rectification, ion selectivity and water flux [252]. (c) All‐atom model of a DNA duplex threading a nanopore drilled between gold plasmonic bow‐tie structures. The red semitransparent surface depicts the isosurface of the plasmonic field intensity , which controls DNA transport and stimulate Raman scattering to report on the DNA sequence [134]. (d) Simulated thermophoretic stretching of ssDNA threaded through a radiatively‐heated orifice of a solid‐state membrane [253]. (e) Translocation velocity of ssDNA through the locally heated nanopore (panel d) as a function of the transmembrane bias. (f) All‐atom model of ssDNA simultaneously captured by the two pores in a hexagonal boron nitride membrane. Two MD simulations of a DNA strand threaded through each pore were coupled via a virtual spring [254]. (g) Flossing of an ssDNA strand in the dual‐nanopore system achieved by alternating application of a 10 V bias across left and right pores. (h) Probability of correctly identifying an ssDNA triplet from the ionic current as a function of the number of reads obtained by flossing the molecule back and forth between the pores. Panels a and b are reproduced from the study by Shankla and Aksimentiev [252], Copyright 2017, *American Chemical Society*, panel c from the study by Belkin et al. [134], Copyright 2015, *American Chemical Society*, panels d and e from the study by Belkin et al. [253], Copyright 2013, *American Chemical Society*, and panels f–h from the study by Choudhary et al. [254], Copyright 2020, *American Chemical Society*, with permission.

Metallic nanostructures illuminated at resonant frequencies can excite surface plasmons, creating extremely large electric fields within nanometers of the metallic surfaces. The resulting enormous electric field gradients can exert large forces on dielectric materials, trapping small plastic beads [255] and even individual biomolecules [256]. We have combined continuum optics simulations with coarse‐grained and all‐atom MD simulations to investigate the possibility of using such plasmonic trapping for controlling the speed of DNA motion through a solid‐state nanopore [134]. The specific system considered in our work included a nanopore in a silicon nitride membrane drilled at the center of a gold bowtie nanostructure, (Figure 6c). The local electric field intensity obtained from the optics calculations was applied as an external potential to DNA molecules in an MD simulation via the Grid‐SMD functionality of NAMD [238, 257]. Switching the plasmonic field on and off produced a stepwise translocation of a DNA molecule under a constant electric field. In addition to trapping DNA, the high electric fields generated by the plasmonic nanostructures also enhance Raman scattering from the nucleotides confined to the hot spot. We have used our MD trajectories to determine conditions for reading the sequence of DNA nucleotides by means of collecting their Raman fingerprints [134].

Incident radiation can also heat local structures that absorb it. To explore how such a phenomenon might be used in DNA sensing applications, we performed simulations that included an absorbing heating element embedded in a silicon nitride membrane in the vicinity of the nanopore. The local heating was modeled by applying a high‐temperature Langevin thermostat to the atoms of the heating element while a room‐temperature Lowe‐Andersen thermostat was applied to water molecules away from the nanopore membrane [253]. The resulting temperature gradient exerted a thermophoretic force on ssDNA, stretching the DNA fragment confined within the nanopore (Figure 6d) and thus enabling radiative modulation of ssDNA translocation velocity under a fixed‐amplitude transmembrane voltage (Figure 6e).

Of all of the enzyme‐free mechanisms we have explored to control nanopore transport, a dual nanopore system provides the most direct method of decoupling the electric field driving DNA translocation from the electric field generating the ionic current readout. The simplest dual nanopore system consists of a single solid‐state membrane where two nanopores are fabricated in close proximity to one another [258]. A DNA strand introduced into the *cis* chamber can be double‐captured by the system of two pores, which increases the duration of nanopore transport by several orders of magnitude in comparison to that of a single nanopore capture [258]. A dual nanopore system can also be manufactured to allow for independent control over the voltage bias applied to each nanopore [259]. When equal transmembrane voltage is applied across both pores, the total force applied on the captured DNA strand by the applied electric field is close to zero. At the same time, the transmembrane bias will generate the ionic current through each nanopore of usual magnitude. By introducing a slight imbalance in the transmembrane biases, the DNA strand can be driven towards either nanopore, a process we modeled using two coupled all‐atom MD simulations [254] (Figure 6f) and a custom coarse‐grained model [258]. Alternating the imbalance in the applied biases allows the molecule to be flossed back and forth, enabling repeat current readout from the same fragments of the DNA molecule (Figure 6g). Repeating the ionic current readout improves the signal‐to‐noise ratio, dramatically increasing accuracy of DNA sequence determination (Figure 6h).

## 7 Analysis of MD Trajectories

The fundamental output of an all‐atom MD simulation is a trajectory, a high‐dimensional record of the 3D coordinates and velocities of every atom in the ensemble over time. To render these raw data scientifically actionable, they must be transformed into physically meaningful parameters that characterize the nanopore transport and enable direct comparison with experimental observables. Operating with a temporal resolution on the order of picoseconds, MD simulations track molecular displacement and velocity at a frequency approximately four to six orders of magnitude higher than the microseconds scale of a typical nanopore measurement. This extreme sampling frequency allows computational models to probe ultrafast transport phenomena that remain elusive to laboratory hardware.

This section reviews the methodologies developed to extract macroscopic signals from microscopic trajectories. We begin with the calculation of ionic current, selectivity, and water flux, detailing the transition from individual atomic counts to steady‐state transport metrics. We then describe the procedures for measuring capture rates and dwell times, which define the kinetics of analyte‐pore interactions. Finally, we introduce methods for measuring the effective forces and torques to provide direct mechanistic insight into the physical drivers of translocation, which are often difficult, if not impossible, to access through experiment alone.

### 7.1 Ionic Current, Selectivity, and Water Flux

To calculate the ionic current from an all‐atom MD simulation, the most common approach is to track the net displacement of charge species along the pore axis [37, 260]. To compute the current for specific ion species, the coordinates of all atoms are typically recorded at high frequency, often every 1 to 5 ps. The instantaneous ionic current I(t) is derived from the net displacement of all charged species during a sampling interval Δt [37]:

(1) $$I\left(t\right)=\frac{1}{\Delta t\;L_{z}}\sum_{i=1}^{N}q_{i}\;\left[z_{i}\left(t+\Delta t\right)-z_{i}\left(t\right)\right],$$

where $q_{i}$ is the charge and $z_{i}$ is the coordinate of atom i and $L_{z}$ represents the length of the sampling volume along the pore axis. While $L_{z}$ can encompass the entire simulation box, it is often convenient to restrict the summation to the channel's interior, Figure 7a, to focus specifically on the transport dynamics within the nanopore and reduce the thermal noise.

**FIGURE 7:**
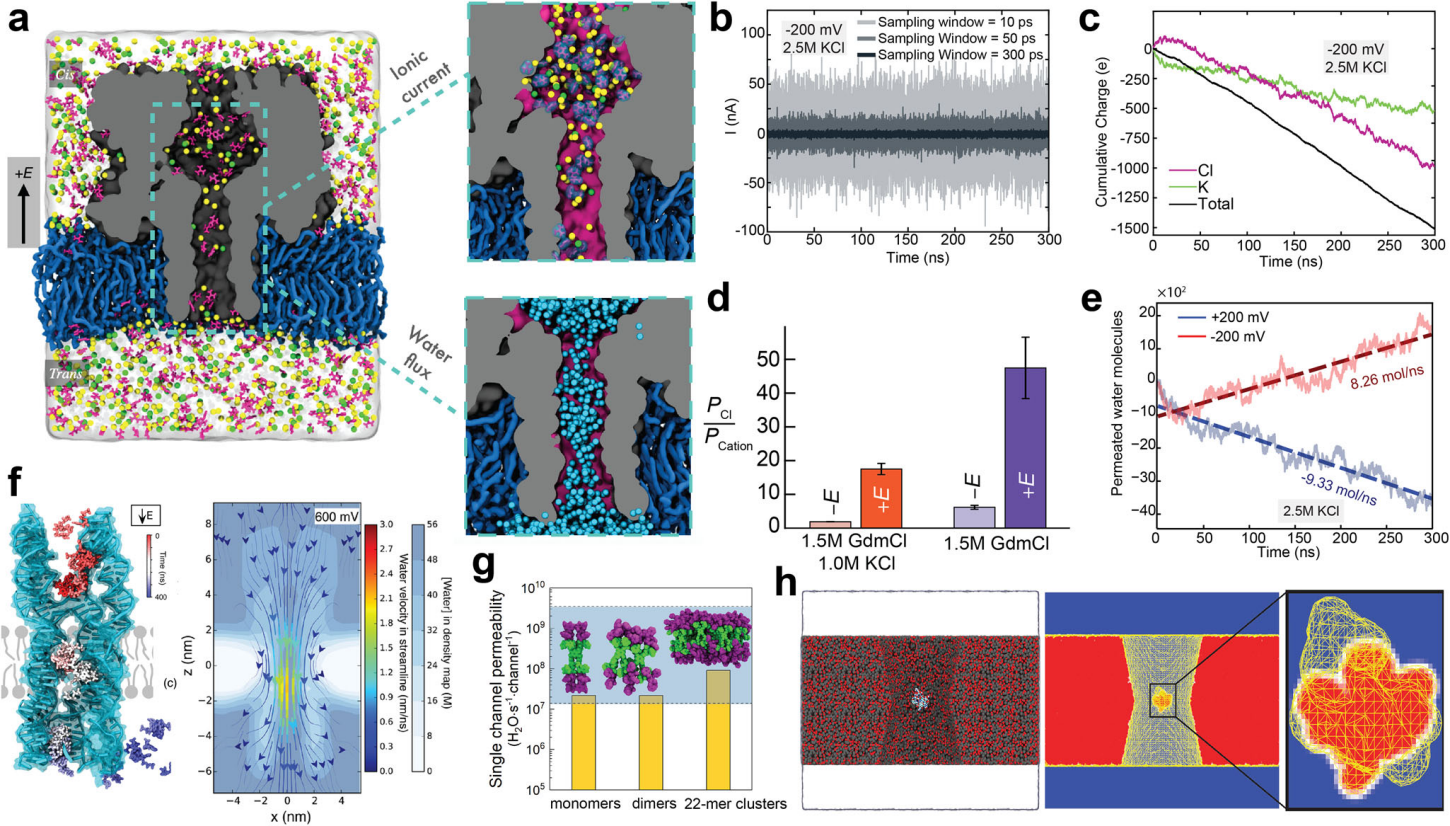
Ionic currents and water flux analyses. (a) Cut‐away view of α‐hemolysin (grey) embedded in a lipid membrane (blue), submerged in electrolyte. The ionic current and water flux are calculated by summing up displacement of the molecules over the MD trajectory. (b) Ionic current through α‐hemolysin over the course of an MD trajectory measured using several sampling intervals [261]. (c) Charge flux through the α‐hemolysin nanopore, calculated as the cumulative integral of the instantaneous current shown in panel b  [261]. The slope of each line corresponds to the average current. ± (d) Simulated selectivity of α‐hemolysin [261]. (e) Water flux through α‐hemolysin over the course of two MD trajectories [261]. (f) Electric field‐driven transport of ATP through a DNA nanopore [180]. (Left) Time‐lapse illustration of a 400 ns MD trajectory showing an ATP molecule passing from one side of the pore to the other through the central pore. (Right) Average local density (grayscale) and velocity (streamlines) of water during the 400 ns MD simulation at 600 mV. (g) MD‐derived average water permeability per PAH channel of a lipid bilayer membrane containing arrays of PAH monomers, highly conductive dimers, and a 22‐mer cluster [262]. The simulated permeability values fall within the experimentally measured permeability range (shaded area). (h) All‐atom representation of a solid‐state nanopore containing a protein within its constriction and the corresponding SEM representation of the system, with a close‐up view highlighting the region near the protein [248]. A heatmap slice through the three‐dimensional map of local conductivity is shown, which, together with the boundary conditions, defines the system for ionic current calculations. Blue and red regions correspond to bulk ion conductivity and zero conductivity, respectively. The yellow wireframe indicates an isosurface where the local conductivity equals 50% of the bulk value. Panels c–e are reproduced from the study by Mehrafrooz et al. [261], Copyright 2024, *American Chemical Society*, panel f from the study by Song et al. [262], Copyright 2020, *Springer Nature*, panel g from the study by Yoo and Aksimentiev [180], Copyright 2015, *American Chemical Society*, and panel h from the study by Wilson et al. [248], Copyright 2019, *American Chemical Society*, with permission.

Because MD coordinates are recorded at picosecond intervals, the resulting instantaneous current is characterized by high‐frequency thermal noise and is inherently dependent on the sampling window Δt, as illustrated in Figure 7b. Despite this transient volatility, the choice of Δt does not influence the long‐term average current values. To extract a statistically meaningful average current at a given bias, the instantaneous values are first integrated to produce a cumulative current curve, as shown in Figure 7c. A linear regression fit applied to the slope of this cumulative curve provides a robust estimate of the steady‐state ionic current.

Several procedural considerations are required to ensure the accuracy of this coordinate‐based method. To prevent systematic errors introduced by the global drift of the simulation box, the nanopore system must be aligned and recentered to its initial coordinates before computing displacements. Furthermore, in simulations utilizing wrapped coordinates, special care must be taken to account for periodic boundary jumps. If an ion crosses a periodic boundary between t and t+Δt, the displacement must be unwrapped to avoid unphysical spikes and ensure a continuous trajectory for the current calculation. Finally, it is important to note that absolute ionic current values obtained from all‐atom MD simulations frequently deviate from experimental measurements due to forcefield inaccuracies [263]. To improve the quantitative agreement between simulation and experiment, the raw MD current is often scaled by the ratio of the experimental bulk electrolyte conductivity and the computationally determined bulk conductivity. Note that many ionic current metrics, such as the ratio of the current at positive and negative bias polarities, that is, rectification, or the ratio of blockade and open pore currents are insensitive to systematic forcefield artifacts and can be directly compared to corresponding experimentally measured quantities [236, 261, 264]. Finally, ionic current calculations have been shown to be largely insensitive to the use of HMR [221] and the associated 4 fs timestep, with negligible differences observed for the OmpF porin channel at low applied voltages [222].

By applying Equation (1) to individual ion species, all‐atom MD enables direct decomposition of the total ionic current into species‐specific contributions (Figure 7c). Species‐resolved currents can be used to compute the ionic selectivity (S), defined as the magnitude of the ratio of anionic to cationic current measured under the same applied electric field:

(2) $$S=\left|\frac{I_{\mathrm{anion}}}{I_{\mathrm{cation}}}\right|.$$

A value of S=1 indicates a non‐selective nanopore, where both ionic species contribute equally to transport. Deviations from unity reflect preferential conduction arising from surface charge, electrostatic interactions, or specific binding motifs within the pore. A ratio S<1 corresponds to cation selectivity, whereas S>1 indicates anion selectivity. In practice, the selectivity of a biological nanopore depends on both the absolute ion concentration and the polarity of the transmembrane bias (Figure 7d). We previously found MD‐derived nanopore selectivity to broadly match the values derived from experimental reverse potential measurements [261].

Complementary to the selectivity ratio, the relative ionic current ($I_{\mathrm{rel}}$) quantifies the imbalance between cationic and anionic contributions:

(3) $$I_{\mathrm{rel}}=I_{\mathrm{cation}}-I_{\mathrm{anion}}.$$

Because oppositely charged ions move in opposite directions under an applied electric field, any imbalance in their flux generates a net ion transport. This net flux of ions transfers momentum to the surrounding solvent and gives rise to electro‐osmotic flow (EOF) [265]. Notably, the EOF flux was found to scale approximately linearly with the relative ionic current, with a proportionality factor that reflects the number of water molecules associated with the hydration shells of the dominant charge carriers [261].

The flux of water through a nanopore can be computed using the same displacement‐based formalism of the ionic current calculation (Figure 7e). Just like with the ionic current, under steady‐state conditions, the specific choice of sampling region along the pore axis does not affect the time‐averaged flux, only the statistical error of the flux determination. An alternative method to quantify water transport tracks the center‐of‐mass (CoM) displacement of all water molecules along the pore axis using an unwrapped trajectory. The drift velocity of the water CoM, combined with the bulk water density and the cross‐sectional area of the simulation box, yields the volumetric flux. It is important to note that ionic current and the EOF can be sensitive to the forcefield choice. Systematic comparisons of CHARMM36, Amber‐ff14SB, and Amber‐ff19SB have shown that while ion selectivity and electroosmosis direction are generally consistent across forcefields, quantitative differences in conductance and magnitude of electroosmotic flow can arise, becoming particularly significant for narrow pores such as MspA [266]. It is therefore advisable to validate the chosen forcefields against experimental conductance and ion selectivity data, if those are available.

Beyond macroscopic transport metrics, the MD method can characterize the local fluid environment in a spatially resolved manner. For such analysis, the simulation volume is partitioned into a three‐dimensional grid of rectangular elements (typically 2×2×2 Å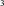) to compute local water (and ion) velocity and density fields. These velocity vectors are transformed into cylindrical coordinates and averaged over the azimuthal angle to obtain a symmetrized two‐dimensional flow field in the r–z plane [180, 267] (Figure 7f, right). This procedure enables the generation of streamline plots that precisely characterize the local flow pattern in a nanopore. To visualize the trajectory of individual solute's transport, it is convenient to draw a time‐lapse plot where multiple instantaneous conformations of a solute molecule are drawn in the background of a static nanopore structure, and using color to indicate the time course of the permeation trajectory (Figure 7f, left). Such spatially resolved analysis provides a direct view of hydrodynamic coupling between solvent and analyte, which is essential for understanding electro‐osmotic transport in confined geometries.

While water flux in MD simulations is typically quantified by counting molecules crossing a reference plane, experimental validation often relies on permeability measurements. Permeability characterizes the ease with which solvent traverses a nanopore under a hydrostatic pressure or chemical potential gradient. In MD simulations, permeability can be obtained by relating the steady‐state water flux to the applied driving force [268], such as a pressure differential or an osmotic gradient. Converting MD‐derived fluxes into permeability coefficients enables direct comparison with laboratory measurements from osmotic swelling or filtration experiments [138, 262, 269] (Figure 7g).

### 7.2 Minimizing Uncertainty of Ionic Current Determination

Measuring nanopore ionic current using the all‐atom MD method traditionally relies on quantifying ion displacement through the nanopore [37, 126, 136, 231]. While conceptually straightforward, this brute‐force approach suffers from substantial thermal and counting noise. Even under high transmembrane bias, resolving picoampere‐scale currents requires microsecond‐scale trajectories to average over stochastic ion crossings. Such requirements make it computationally expensive to extract subtle differences in conductance arising from small structural changes. To overcome this limitation, several theoretical frameworks have been developed to predict ionic current directly from atomic coordinates, thereby decoupling the physical translocation process from the determination of its electrical signal [248, 270]. These approaches eliminate counting noise by replacing explicit ion flux with continuum conductivity calculations based on the instantaneous geometry of the pore and the analyte.

An early implementation estimated nanopore resistance (R) by discretizing the simulation volume into cylindrical segments, that were further subdivided into 1 Å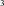 voxels. Each voxel was assigned a local conductivity (σ) as a function of its distance to the nearest molecular surface, and the total ionic current (I) was computed via Ohm's law (I=V/R) by treating the system as a network of resistors. This idea was formalized into the steric exclusion model (SEM) [270], which assumes that local electrolyte conductivity depends on the bulk conductivity modulated by steric exclusion from nearby non‐solvent atoms [27, 271]. By discretizing the nanopore into resistance elements, the SEM solves the resulting resistor network to determine the blockade current. Subsequent refinements extended SEM to complex nanopore geometries by constructing an atomically resolved three‐dimensional conductivity map (Figure 7h) [248]. The resulting 3D resistor network is solved numerically using finite‐element methods to obtain steady‐state ionic current. Compared to brute‐force MD calculations, SEM is orders of magnitude more computationally efficient while maintaining high accuracy across different pores and analytes [248].

Because SEM is inherently insensitive to thermal fluctuations and counting noise, it can substantially increase the precision of blockade current determination. Instead of computing the current directly from a long MD trajectory, one can first generate a representative ensemble of conformations illustrating a translocation event using a biased MD approach. The ionic current is then evaluated for each static configuration using SEM, yielding noiseless blockade levels determined solely by the instantaneous three‐dimensional configuration of the simulation system. This approach was used to reconstruct blockade traces of peptide translocation through aerolysine nanopore [89], cytochrome c [234] and knotted proteins [239] translocating through silicon nitride nanopores, and SBD2 proteins trapped within ClyA nanopore [272]

Beyond thermal noise reduction, the SEM framework also enables high‐throughput in silico mutagenesis and chemical screening. For example, in the studies of peptide discrimination by the aerolysin nanopore [89, 273], the effect of amino acid substitutions was evaluated directly through the SEM calculation by modifying the peptide's sidechain identities while keeping the global molecular configuration constant. By swapping residues in silico, one could isolate the contributions of individual mutations to the blockade signal without introducing conformational noise that would be present if one were to perform nanopore translocation simulations for each variant. By separating translocation simulations and ionic current determination, molecular modeling of nanopore transport transforms from providing qualitative description of the translocation processes into a predictive, quantitative tool for rational nanopore design.

### 7.3 Capture Rate and Dwell Time

Analyte transport is experimentally characterized by the capture rate, which refers to the frequency of molecules entering the pore, and the dwell time, which refers to the duration of their residence within the nanopore volume (Figure 1d). While stochastic capture events typically occur in experiments on a timescale far greater than that of an MD run, enhanced sampling simulations can be used to probe factors that govern these kinetics. Analyte capture is primarily governed by analyte concentration, pore geometry, and the entropic penalties associated with confinement [274, 275, 276], and can be further influenced by the electric‐field landscape near the pore [277], as well as electrostatic and hydrodynamic forces [278]. Other physical effects, such as dielectrophoresis [279, 280], thermophoresis [281], and plasmonic heating [144], can modulate the capture behavior. Given the complexity of these combined effects, computational analyses of analyte capture have become essential for resolving the underlying mechanisms and for characterizing “failed” collision events that are mostly invisible in experiments but are critical for understanding nanopore sensing efficiency.

In MD simulations, to increase the probability of analyte capture, the analyte can be restrained to remain coaxial with the nanopore axis while allowing rotational freedom and axial motion [144, 234, 282]. To obtain statistically meaningful kinetic parameters, multiple simulations can be performed under the same applied bias. From these trajectories, the mean dwell time, also referred to as the average translocation time, and the average capture rate can be determined. For example, in MD simulations of DNA capture and translocation through a graphene nanopore [282], each system was initialized with the DNA positioned coaxially above the pore at a pre‐defined distance (Figure 8a). Capture time was measured as the time elapsed from the moment the DNA molecule was free to move along the nanopore axis and the time when the center of mass of the leading DNA base pair crossed the graphene plane for the last time (the molecule could make multiple attempts to enter the pore). The average capture rate was computed as the average of the inverse capture time. In this particular system, the non‐monotonic dependence of the capture rate was explained by the water compression effect [282]. The dwell time was defined as the subsequent interval between that final crossing event for the leading base pair and the moment when the trailing base pair passed through the membrane plane (Figure 8b). Given the small volume of an all‐atom MD system, the simulated capture rates are much larger than those observed experimentally. Unrestrained capture simulations, albeit coarse‐grained, can reproduce the experimental capture rates, after accounting for the concentration difference [283].

**FIGURE 8:**
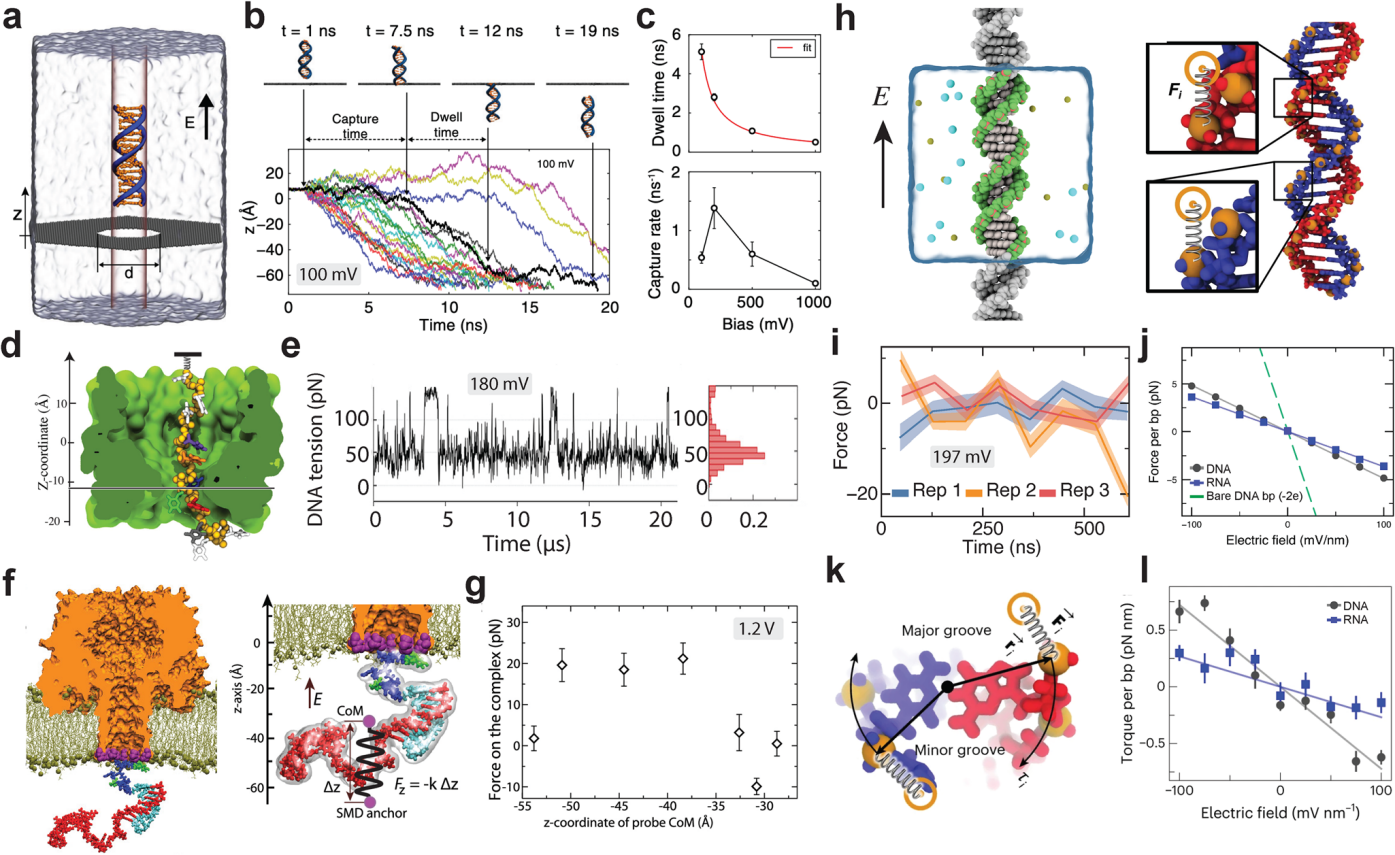
Capture rate, dwell time, and effective force. (a) Simulation setup of the DNA‐graphene nanopore system [282]. The phosphorus atoms of the DNA fragment are confined within a 1.88 nm diameter cylinder coaxial with the nanopore axis. (b) *Z*‐coordinate of the leading DNA base pair versus time in twenty independent MD simulations performed under a 100 mV transmembrane bias [282]. The graphene membrane is positioned at z=0. Snapshots show representative configurations of the DNA fragment along the highlighted translocation trajectory (black). During the first 1 ns of each simulation, the z‐positions of the DNA phosphorus atoms were restrained to their initial values. (c) Voltage dependence of the average dwell time (top panel) and average capture rate (bottom panel) obtained from MD simulations [282]. The red line indicates a 1/V fit. (d) System for measuring the effective force on ssDNA in a truncated model of the MspA nanopore [42]. A harmonic potential applied to one end of the DNA prevents its nanopore translocation. (e) Effective force on ssDNA in MspA under a 180 mV transmembrane bias measured by the extension of the harmonic spring potential [42]. (f) System for measuring the capture forces on a DNA–PNA probe near the entrance of an α‐HL nanopore. Right snapshot illustrates the SMD method for determining the effective force on the probe [280]. (g) Effective force on the construct under a 1.2 V bias versus the *z*‐coordinate of the complex' CoM (shown in panel f) [280]. (h) System for measuring the effective force of the applied electric field on duplex DNA [284]. (i) Time series of effective force on DNA for the system shown in panel h for three replicas under a 197 mV bias. (j) Electric field dependence of the effective force acting on a DNA or RNA duplex in solution. (k) Illustration of the method used to measure the torque acting on a DNA duplex from harmonic restraints applied to phosphorus atoms. (l) Electric field dependence of the effective torque acting on a DNA or RNA duplex in solution. Panels a–c are reproduced from the study by Wilson and Aksimentiev [282], Copyright 2018, *American Chemical Society*, panels d–e from the study by Bhattacharya et al. [42], Copyright 2016, *American Chemical Society*, and panels f–g from the study by Tian et al. [280], Copyright 2013, *American Chemical Society*, with permission. Panels h, j, k, and l are reproduced from the study by Maffeo et al. [284], Copyright 2023, *Springer Nature*, with permission.

### 7.4 In Silico Measurement of the Effective Forces and Torques

Understanding the mechanical forces acting on an analyte is essential for deciphering translocation physics and optimizing sensor resolution, yet traditional experimental tools face significant hurdles. In the laboratory, the gold standards for force measurement are atomic force microscopy (AFM), which uses a sharp probe on a flexible cantilever to “feel” and pull on samples [285], and optical tweezers, which employ a highly focused laser beam to trap dielectric beads attached to a molecule and measure piconewton‐scale forces via bead displacement [286]. While these methods are indispensable for studying molecular elasticity in solution, they are difficult, but not impossible [287, 288], to integrate with nanopore measurement because of the physical size mismatch between the AFM tip or the micron‐sized beads and the nanopore, as well as their interactions with the membrane. An MD simulation is not subject to these limitations and can be used for direct calculation of the total effective force and torque experienced by an analyte.

While an MD program internally evaluates instantaneous forces acting on each and every atom at every simulation time step, using such direct force outputs for effective force determination is impractical because of large stochastic fluctuations and a near‐zero time‐averaged force. Instead, the effective force on an analyte can be conveniently measured by restraining displacement of an analyte within the nanopore using a harmonic spring potential [175]. Figure 8d depicts one such situation where one end of the analyte (here, a DNA strand) is harmonically restrained to a point above the nanopore constriction whereas the other end is threaded all the way through the nanopore. In equilibrium, the effective force on the DNA is balanced by the force of the harmonic restraint, which is the product of the restrained atom's displacement and the spring constant. This method was used to quantify the electro‐osmotic screening of the DNA charge in solid‐state nanopores [175], the temperature [144] and secondary structure [289] dependence of the effective force, as well as the force on ssDNA [42] (Figure 8e) or a charged polypeptide chain [290] in MspA. The in silico measurements of the effective force are not limited to placement of the analyte within the nanopore. Thus, we have used the SMD method to quantify forces acting on the DNA–PNA hybrids (Figure 8f), explaining the unexpected capture of a charged probe under a “wrong” polarity bias and revealing the role of dielectrophoretic forces (Figure 8g) in promoting such capture [280]. The instantaneous values of the effective force typically exhibit large fluctuations. Thus, obtaining a statistically significant force value either requires a long continuous simulation (Figure 8e) or multiple replicate simulations of the same system (Figure 8i).

In silico measurement of the effective force can also reveal the physical factors responsible for its specific value. In our simulations of the effective force on a DNA duplex, each phosphorus atom was harmonically restrained to its template coordinate (Figure 8h). Summing over all restrained atoms' displacement produced the value of the force that was considerably smaller than the nominal force of the electric field on the bare DNA charge (Figure 8j). Previously, we have attributed such an effective charge reduction to the drag of the solvent flow [175], which in turn is caused by the cloud of counterions surrounding the DNA duplex and rendering the nearby fluid electrically charged. Could this electro‐osmotic flow generate torque on a helical DNA or RNA duplex due to their turbine‐like shape? To determine the torque from an MD simulation, we can harmonically restrain the duplex' atoms within a plane normal to the direction of the electric field and use the definition of torque ($\tau =\vec{r}\times \vec{F}$) to gauge the rotational forces (Figure 8k) [284]. Indeed, we observe a small but statistically significant torque acting on a duplex in an electric field (Figure 8l). Additional simulations performed without such restraints directly confirmed the presence of torque as the DNA duplex was observed to rotate unidirectionally in spite of considerable stochastic fluctuations. The torque acting on a DNA duplex in a nanopore was predicted to supercoil DNA [284], a computational prediction that was confirmed by experiment [291].

## 8 Outlook

Over the past two decades, the effective capability of the all‐atom MD method, assessed by attainable system size, trajectory length, and aggregate sampling throughput, has increased by orders of magnitude, approaching a million‐fold improvement when combining advances in hardware acceleration, parallel scalability, and algorithmic efficiency [292, 293]. In 2005, a ten‐nanosecond nanopore simulation represented the limit of feasibility; today, microsecond‐scale trajectories are routine, and millisecond sampling is no longer unthinkable with specialized architectures and exascale resources pushing even further [294]. This dramatic expansion has not merely extended trajectories; it has redefined what questions can be asked, narrowing the gap between in silico and in vitro observations. If such growth continues, where will nanopore simulations stand in 10 years? The answer will likely be shaped by three converging frontiers: improved forcefield fidelity, mature multiresolution strategies, and the integration of machine learning for both dynamical acceleration and signal interpretation.

Although further improvements in forcefield accuracy remain essential for achieving fully predictive nanopore simulations, the field is uniquely positioned to address this challenge. Unlike many biomolecular systems, nanopores provide rich, quantitative single‐molecule observables—for example, ionic conductance, current–voltage dependence, dwell‐time distributions, and voltage‐dependent transport—that are directly coupled to atomic‐scale structure and dynamics. These experimental data offer stringent and continuous benchmarks against which atomistic models can be tested and iteratively refined. In this sense, nanopore measurements are not merely targets for simulation validation; they constitute powerful calibration platforms capable of driving forcefield improvement. As simulation throughput continues to expand,t a feedback loop between experiment and computation may transform nanopore research into a model system for co‐evolving accuracy, where predictive molecular dynamics is progressively refined by high‐resolution electrical measurements.

Multiresolution simulation frameworks [295] will likely define the next decade of nanopore modeling. Purely all‐atom descriptions, even in the exascale era, remain fundamentally constrained by the trade‐off between temporal reach and chemical detail. Yet, nanopore phenomena are inherently multiscale: atomic rearrangements at the constriction govern ion dehydration and signal fluctuations, while polymer conformational entropy and membrane mechanics extend over tens to hundreds of nanometers. The central challenge is therefore not merely scaling brute‐force atomistic simulations, but orchestrating a seamless resolution exchange across these regimes. Emerging adaptive‐resolution schemes, hybrid particle–continuum electrostatics, and regionally refined forcefields offer a conceptual path forward, but their deployment in driven, nonequilibrium transport remains a significant hurdle. Robust coupling must preserve thermodynamic consistency, momentum transfer, and fluctuation spectra under applied voltage conditions. A promising direction is dynamic resolution allocation, in which the pore constriction and translocating analyte are retained at full atomistic detail while distal reservoirs and membranes are represented through coarse‐grained or learned effective potentials. Such approaches, combined with enhanced sampling, could finally extend physically faithful simulations to the millisecond timescales of laboratory measurements without sacrificing the chemical specificity that defines nanopore selectivity.

Parallel to these physical modeling advances, machine learning will increasingly transform simulated nanopore data from a validation tool into a generative engine for sequencing and biomarker discovery. High‐throughput MD can now produce vast libraries of labeled trajectories in which the ground‐truth identity, orientation, modification state, and conformational ensemble of each analyte are precisely known. From these trajectories, one can compute synthetic ionic currents, force profiles, hydration signatures, and fluctuation spectra under systematically varied voltages and chemistries. Such datasets provide an unprecedented substrate for training models that learn the nonlinear mapping between microscopic structure and macroscopic electrical signal, free from the ambiguities and experimental noise that obscure purely empirical training. Crucially, machine learning can operate bidirectionally: accelerating dynamics via learned forcefields or collective variables, and decoding signals through physics‐informed architectures that embed conservation laws and electrostatic constraints. The convergence of simulation and learning thus suggests a closed‐loop paradigm in which models trained on synthetic data guide pore design, predict discriminative signal features for rare modifications, and prioritize experimental conditions, while experimental measurements iteratively recalibrate both forcefields and inference networks. In this integrated framework, nanopore platforms may evolve from passive sensors into adaptive molecular interrogators, where predictive simulation and data‐driven recognition co‐develop toward real‐time, single‐molecule diagnostics.

## Conflicts of Interest

The authors declare no conflicts of interest.

## Data Availability

Data sharing not applicable to this article as no datasets were generated or analyzed during the current study.
